# Subcellular thiol functional group distribution in *Geobacter sulfurreducens* determined by Hg L_*III*_-edge EXAFS

**DOI:** 10.3389/fmicb.2025.1728775

**Published:** 2026-02-04

**Authors:** Fanchao Meng, Ulf Skyllberg, Yangyang Li, Shusaku Hayama, Erik Björn, Yu Song

**Affiliations:** 1 Key Laboratory of Pollution Ecology and Environmental Engineering, Institute of Applied Ecology, Chinese Academy of Sciences, Shenyang, China; 2 University of Chinese Academy of Sciences, Beijing, China; 3 Department of Forest Ecology and Management, Swedish University of Agricultural Sciences, Umeå, Sweden; 4 Diamond Light Source, Didcot, United Kingdom; 5 Department of Chemistry, Umeå University, Umeå, Sweden

**Keywords:** cellular thiols, mercury biogeochemistry, speciation and bioavailability, synchrotron X-ray absorption spectroscopy, uptake and methylation

## Abstract

Mercury (Hg) is a global environmental concern due to its microbial conversion to methylmercury (MeHg), a potent neurotoxin that bioaccumulates in food webs and poses risks to ecosystems and human health. Thiol functional groups (RSH) play an important role in controlling Hg(II) speciation and bio-uptake in methylating bacteria, yet the spatial distribution and density of these thiols within cells remain largely unknown. We isolated subcellular fractions of the Hg methylating bacterium *Geobacter sulfurreducens* in the exponential growth phase, and used Hg L_*III*_-edge EXAFS (Extended X-ray Absorption Fine Structure) to quantify thiols in the extracellular medium, inner and outer membranes, periplasm and cytoplasm. The whole-cell thiol content was determined to be 1.3 × 10^−10^ μmol cell^−1^. The inner membrane contributed 7.1 × 10^−11^ (53%), the outer membrane 1.2 × 10^−11^ (9%), the periplasm 3.6 × 10^−11^ (27%) and the cytoplasm 1.5 × 10^−11^ μmol cell^−1^ (11%). The extracellular fraction contributed an additional 5.7 × 10^−11^ μmol cell^−1^, corresponding to 30% of the thiols of the cell culture. Local thiol density (thiols normalized to TOC in individual compartment, RSH/TOC, μmol g^−1^ C) was 36, 450, 140, 600 and 29 μmol g^−1^ C in the cytoplasm, inner membrane, periplasm, outer membrane and extracellular fractions, respectively. EXAFS analyses demonstrate Hg-thiolate coordination across all compartments, with Hg-O/N bonding and elemental Hg^0^ formed at higher Hg loadings. In the periplasm, Hg-disulfide and traces of *β*-HgS were detected. The high thiol density at the membranes, relative to other compartments, may imply they have an important role in the retention and internalization of Hg(II). Periplasmic thiols may modulate Hg(II) transfer between membranes, and cytoplasmic thiols may regulate the intracellular availability of Hg(II) for methylation. This work provides the first compartment-resolved quantification of thiol abundances and densities in a model Hg-methylating bacterium at subcellular level, offering a mechanistic framework for understanding the speciation, bioavailability, and subcellular transformation of Hg(II) with relevance for other soft metals (e.g., Cd, Pb, Zn, Ag, and Cu).

## 1 Introduction

Mercury (Hg) is ranked among the top ten chemicals of major public health concern by the World Health Organization (WHO). Its most toxic and bioaccumulative form, methylmercury (MeHg), poses serious risks to ecosystems and human health. MeHg is primarily produced intracellularly by anaerobic and facultatively anaerobic microorganisms that possess the *hgcAB* gene pair, including iron-reducing, sulfate-reducing, methanogenic, and firmicute bacteria (Gilmour et al., 2013; Hsu-Kim et al., 2013; Parks et al., 2013; Schaefer et al., 2014; Podar et al., 2015; Regnell and Watras, 2019). A critical step in MeHg formation is the microbial uptake of Hg(II), yet the molecular mechanisms underlying the transmembrane transport of Hg(II) remain poorly understood.

In *mer*-resistant bacteria, Hg(II) uptake is mediated by the *mer* operon transporters, including MerC, MerP, and MerT, where thiol groups facilitate Hg(II) binding and transfer (Barkay et al., 2003). However, these *mer*-resistant bacteria are predominantly aerobic and lack the ability to methylate Hg(II). The Hg(II)-methylating bacteria are hypothesized to acquire Hg(II) through both passive diffusion and active transport (Hsu-Kim et al., 2013). Passive uptake is thought to involve neutral Hg(II) complexes such as $\text{HgC}{\text{l}}_{2_{\left(aq\right)}^{0}}$, $\text{Hg}{\left(\text{OH}\right)}_{2_{\left(aq\right)}^{0}}$, $\text{Hg}{\left(\text{SH}\right)}_{2_{\left(aq\right)}^{0}}$ (Gutknecht, 1981; Mason et al., 1996; Benoit et al., 1999a,b, 2001; Hsu-Kim et al., 2013; Zhou et al., 2017) and possibly nanoparticulate HgS forms (Deonarine and Hsu-Kim, 2009; Zhang et al., 2012; Guo et al., 2023). Schaefer and Morel (2009) and Schaefer et al. (2011) demonstrated active uptake in the presence of low-molecular-mass thiols (LMM-RSH), particularly cysteine (Cys), by model methylating bacteria, including *Geobacter sulfurreducens* PCA and *Pseudodesulfovibrio mercurii* ND132 (formerly *Desulfovibrio desulfuricans* ND132).

All these studies introduced LMM thiols at relatively high concentrations under laboratory conditions, which will alter extracellular Hg(II) speciation and may affect bacterial metabolism as well (Adediran et al., 2019). By use of mass spectrometry, Adediran et al. (2019) demonstrated that *G. sulfurreducens* synthesizes and secretes LMM thiols (primarily cysteine) at concentrations up to 100 nM that can significantly influence extracellular Hg speciation, due to the high affinity between Hg(II) and thiol groups (Liem-Nguyen et al., 2017; Skyllberg, 2008; Song et al., 2018, 2020). Gutensohn et al. (2023a,b) showed that *G. sulfurreducens* further transforms and metabolizes LMM thiols (specifically metabolic conversion of cysteine to penicillamine) which have a strong influence on Hg(II) uptake and methylation.

In addition to extracellular thiols, considerable research has focused on thiol groups at the cell surface (outer membrane of Gram-negative bacteria). Quantification methods of cell surface thiols typically involve derivatization followed by detection using electrochemical techniques (Mishra et al., 2017; Yu et al., 2014), fluorescence spectroscopy (Joe-Wong et al., 2012; Mishra et al., 2017; Rao et al., 2014; Wang et al., 2016) or X-ray absorption spectroscopy (XAS), such as Hg L_*III*_-edge extended X-ray absorption fine structure (EXAFS) spectroscopy (Song et al., 2020). While electrochemical and fluorescence-based techniques offer nanomolar sensitivity, they are prone to selectivity issues and matrix interference. In contrast, Hg EXAFS enables element-specific, matrix-independent detection, though it requires synchrotron access and has relatively higher detection limits.

Several studies have suggested that membrane thiols promote Hg(II) uptake and transformation in model methylating bacteria, *G. sulfurreducens* and *P. mercurii* (An et al., 2019; Dunham-Cheatham et al., 2015; Lin et al., 2014; Mishra et al., 2017; Wang et al., 2016; Zhao et al., 2017). Adediran et al. (2019) proposed a surface complexation model wherein Hg(II) forms ternary complexes with cell surface thiols and extracellular ligands (L), possibly facilitating Hg(II) internalization on *G. sulfurreducens*. In support of this view, an EXAFS study of *G. sulfurreducens* demonstrated that 5% of membranes thiols (Mem-RSH, mixture of inner and outer membranes) formed bis-thiolate complexes, Hg(Mem-RS)_2_; while the remaining 95% were spatially separated, enabling the formation of ternary complexes, Hg(Mem-RS)L (Song et al., 2020). In a follow-up study, using the same EXAFS methodology, Gutensohn (2023) demonstrated that 42% of outer membrane thiols and 96% of inner membrane thiols participate in ternary complexation. In contrast, some studies implied that membrane thiols may act as a barrier for uptake by sequestering Hg(II) at the cell surface suppressing Hg(II) internalization (Graham et al., 2012; Liu et al., 2016; Hu et al., 2013). Moreover, one study indicated that membrane thiols had no significant impact on Hg(II) uptake (Thomas et al., 2020).

While membrane thiols have been quantified and their interactions with Hg(II) examined using EXAFS, most studies have examined either the outer membrane alone (Mishra et al., 2017) or a mixture of inner and outer membranes (Song et al., 2020). So far little is known about thiol concentrations and interactions with Hg(II) in other subcellular fractions, such as the cytoplasm and periplasm, despite their potential roles in controlling Hg cellular uptake and methylation. To address this knowledge gap, we present the first quantitative estimates of thiol concentrations across *G. sulfurreducens* subcellular compartments such as cytoplasm, periplasm, membranes (calculated as the difference between whole-cell, cytoplasm and periplasm) using Hg L_*III*_-edge EXAFS spectroscopy, alongside measurements of dry mass, total organic carbon (TOC), and total sulfur (TS) in each cellular-associated fraction. The purpose is to provide a comprehensive view of thiol functional group densities and their distribution at the subcellular level to lay the basis for the mechanistic understanding of Hg(II) uptake and transformation in association with methylating bacteria. This information is in turn necessary to suggest measures to mitigate the adverse effects of MeHg production in different environments.

## 2 Materials and methods

### 2.1 Materials

All solutions used in this study were prepared using degassed Milli-Q water (18 MΩ· CM; Millipore) inside an N_2_-filled glovebox. The degassed water was produced by boiling Milli-Q water under a nitrogen gas purge for 2 hours to remove oxygen. All solutions were either freshly prepared or stored overnight in the glovebox prior to use.

A Hg(II) stock solution (100 g L^−1^) was prepared by dissolving mercuric nitrate monohydrate [Hg(NO_3_)_2_·xH_2_O, CAS: 7783-34-8, Aladdin, Shanghai] in 12% nitric acid. Working solutions at concentrations of 0.01, 0.1, 1 and 10 g L^−1^ were prepared from the stock solution.

### 2.2 Bacterial cultures

*Geobacter sulfurreducens* PCA (Caccavo et al., 1994) was purchased from DSMZ and cultivated under an N_2_ atmosphere at 30 °C and pH 6.8 (± 0.05) in “standard” growth medium as described by Schaefer and Morel (Schaefer and Morel, 2009), with selenium omitted to prevent interference in Hg L_*III*_-edge EXAFS measurements. The medium (pH 6.8) contained 1 g L^−1^ yeast extract, 40 mM sodium fumarate, 10 mM sodium acetate, 10 mM MOPS, 5 mM NH_4_Cl, 1.3 mM KCl, 0.25 mM MgSO_4_, 0.17 mM NaCl, 80 μM nitrilotriacetic acid, 50 μM NaH_2_PO_4_, 8.8 μM CaCl_2_, 1 mg L^−1^ trace metals which contained 30 μM MnCl_2_, 4.2 μM CoCl_2_, 3.6 μM FeSO_4_, 3.5 μM ZnSO_4_, 0.4 μM NiCl_2_, 0.4 μM Na_2_MoO_4_, 0.04 μM CuSO_4_, and 1 mg L^−1^ resazurin. All media and buffers were prepared in acid-cleaned serum bottles, boiled under a continuous N_2_ purge, and sterilized by autoclaving.

Cultures (1−1.5 L) were harvested during the exponential growth phase (OD_600_: 0.5–0.8, corresponding to OD_660_: 0.4–0.7; Supplementary Figure S1) by centrifugation at 5.000 g for 10 min at 4 °C. Cell density was determined using a hemocytometer (DB-180M Series Microscope). Cultures collected after 2 days (OD_600_ ~0.5) were classified as middle-exponential phase, and those after 3 days (OD_600_ ~0.8) as late-exponential phase.

To assess whether cytoplasmic thiol concentrations respond to nutrient levels, additional sodium fumarate or sodium sulfate was added to the growth medium to create a high-carbon (“High C”; 3× fumarate) and high-sulfate (“High S”; 10× sulfate) growth medium.

### 2.3 Subcellular fractions isolation

**Figure 1:**
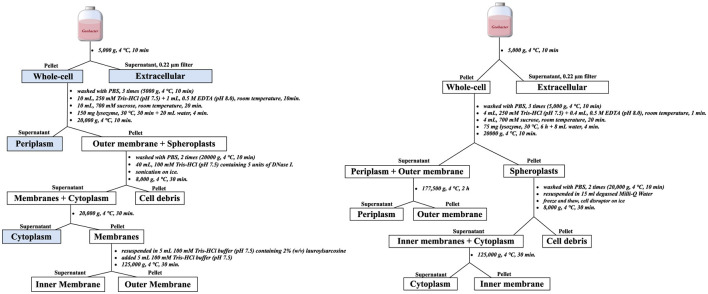
Schematic illustration of the subcellular fractionation procedure. **Left**: Protocol 1, adapted from established methods for *G. sulfurreducens* (Inoue et al., 2010; Jahan et al., 2019), with EXAFS analysis performed on samples outlined in blue. **Right**: Protocol 2, a modified version of Protocol 1 incorporating adjustments based on procedures from Wang et al. (2016) and An et al. (2019).

The subcellular fractionation workflow was performed using two different protocols, as illustrated in Figure 1. Protocol 1 was adapted from established methods for isolating protein subcellular fractions in *G. sulfurreducens* (Inoue et al., 2010; Jahan et al., 2019). Protocol 2 was a modified version of Protocol 1, optimized for spheroplast preparation, in which the outer membrane and spheroplast were separated earlier in the process (Wang et al., 2016; An et al., 2019).

Extracellular fractions were obtained by vacuum filtration (0.2 μM) of cell culture suspensions, with the filtrate defined as the extracellular component. Cell pellets were rinsed at least twice with degassed phosphate-buffered saline (PBS; pH 7.4; 0.14 M NaCl, 3 mM KCl, 10 mM Na_2_HPO_4_, and 2 mM KH_2_PO_4_) and designated as “whole-cell” samples. A portion of these samples was resuspended in degassed Milli-Q water and subjected to disruption with a cell disruptor (SCIENTZ-IID, Ningbo) to ensure complete lysis and release of all intracellular thiols, including those in the cytoplasm, periplasm, and membranes. The remaining cells were fractionated into subcellular compartments using the two protocols described below.

*Protocol 1:* The whole-cell pellet was initially resuspended in 10 mL of 250 mM Tris-HCl buffer, and 1 mL of 0.5 M EDTA was added. After 10 min of incubation at room temperature, 10 mL of 700 mM sucrose was added, and the mixture was incubated for 20 min at room temperature. Following this, 150 mg of lysozyme was added, and the suspension was incubated at 30 °C for 30 min. Osmotic shock was then induced by adding 20 mL of degassed Milli-Q water for 4 min. The mixture was centrifuged at 20.000 g for 10 min at 4 °C, and the supernatant was collected and designated as the periplasmic fraction. The resulting pellet, consisting of spheroplasts and outer membrane fragments, was washed at least twice with PBS buffer to remove residual lysozyme and sucrose. The washed spheroplasts were resuspended in 40 mL of 100 mM Tris-HCl buffer (pH 7.5) containing 5 units of DNase I and disrupted by sonication on ice. The lysate was centrifuged at 8.000 g for 30 min at 4 °C to remove cell debris. The resulting supernatant was further centrifuged at 20.000 g for 30 min at 4 °C, and the final supernatant was designated as the cytoplasmic fraction. The pellet was resuspended in 5 mL of 100 mM Tris-HCl buffer (pH 7.5) containing 2% (w/v) lauroylsarcosine, followed by the addition of another 5 mL of 100 mM Tris-HCl buffer. The suspension was then centrifuged at 125.000 g for 30 min at 4 °C (Beckman, Ultra-High Speed Centrifuge, L-100XP, Type 90 Ti rotor). The supernatant was collected as the inner membrane, and the pellet was reserved as the outer membrane.

*Protocol 2:* Cell pellets were resuspended in 4 mL of 250 mM Tris-HCl buffer (pH 7.5), followed by the addition of 0.4 mL of 0.5 M EDTA (pH 8.0) to chelate divalent cations within the peptidoglycan layer. After 1 minute of incubation at room temperature, 4 mL of 700 mM sucrose and 75 mg of lysozyme were added. The mixture was incubated at 30 °C for 6 hours. To induce osmotic shock and release periplasmic contents, 8 mL of degassed Milli-Q water was added. The suspension was then centrifuged at 20.000 g for 10 min at 4 °C. The resulting supernatant, containing the periplasmic and outer membrane components, was subjected to ultracentrifugation at 177.500 g for 2 h at 4 °C using a Beckman Type 90 Ti rotor. The pellet was designated as the outer membrane fraction, while the supernatant was collected as the periplasmic fraction. This approach differs from Protocol 1 in that the extended lysozyme treatment allows outer membrane material to partition into the supernatant rather than the pellet. This modification was adapted from spheroplast isolation protocols (An et al., 2019; Wang et al., 2016). The initial 20.000 g pellet, corresponding to spheroplasts, was washed twice with PBS (20.000 g, 4 °C, 10 min) to remove residual lysozyme and sucrose. The washed spheroplasts were resuspended in 15 mL of degassed Milli-Q water, freeze-thawed, and disrupted on ice using an ultrasonic homogenizer (SCIENTZ-IID) to lyse the inner membrane. The lysate was centrifuged at 8.000 g for 30 min at 4 °C to remove cell debris. The supernatant was then ultracentrifuged at 125.000 g for 30 min at 4 °C to separate the cytoplasmic fraction (supernatant) from the inner membrane (pellet).

To minimize sample oxidation during subcellular fractionation, all solutions were prepared using degassed Milli-Q water, and all operations were conducted inside a glovebox, except for centrifugation. Before centrifugation, the tubes were filled with an N_2_ atmosphere. Samples were protected from light by covering the vessels with aluminum foil.

### 2.4 Subcellular characterization

The subcellular dry mass was determined after freeze-drying, calculated as the difference between the measured weight and the theoretical dry weight of the added extractant when present. Total organic carbon (TOC) and total sulfur (TS) contents in subcellular fractions were measured using an elemental analyzer (Vario MACRO cube) with a relative standard deviation (RSD) of 0.5%. Total Hg concentrations were determined by combustion atomic absorption spectroscopy using a direct mercury analyzer (DMA-80, Milestone; detection limit =0.01 ng; RSD =6%). The reference material IAEA-456 (Hg = 77 ± 5 μg kg^−1^) was used and yielded recoveries of 95%–105%.

### 2.5 X-ray absorption spectroscopy analyses

#### 2.5.1 Sulfur K-edge XANES

Sulfur K-edge X-ray Absorption Near Edge Structure (XANES) spectroscopy was used to characterize sulfur speciation in cytoplasmic, periplasmic, extracellular, and whole-cell fractions. Subcellular fraction samples were freeze-dried immediately after the stepwise subcellular isolation process. Sulfur K-edge XANES spectra were collected at Beamline 4B7A in Beijing Synchrotron Radiation Facilities (BSRF), China, equipped with a Si(111) double-crystal monochromator. The storage ring was operated at 2.2 GeV with a ring current of 100 mA. To minimize self-absorption effects, a minimum amount of sample was rubbed onto a sulfur free tape. The sample was mounted in a sample cell and flushed with He. Measurements were conducted at ambient temperature under high vacuum (10^−8^−10^−6^ mbar). The incident X-ray energy was scanned from 2.462 to 2.500 eV with a step size of 0.2 eV.

Reference compounds used for spectral calibration and deconvolution included mackinawite (FeS), metacinnabar (HgS), elemental sulfur (S^0^), cysteine (RSH), dithionitrobenzoic acid (RSSR), dibenzothiophene (RSR), Na_2_SO_3_, sulfone, sulfonate, and Na_2_SO_4_. Data processing, including normalization and linear combination fitting (LCF), was performed using the software Larch (Newville, 2013). The pre-edge background was subtracted by fitting a linear function within the energy range of -30 to -10 eV relative to E_0_. For post-edge normalization, a polynomial function was applied to fit the baseline in the energy range of 20−40 eV relative to E_0_.

#### 2.5.2 Hg L_*III*_-edge EXAFS

Hg L_*III*_-edge EXAFS was employed to characterize mercury coordination environments and quantify thiol groups in cytoplasmic, periplasmic, extracellular, and whole-cell fractions, following established protocols (Song et al., 2018, 2020). After isolation, subcellular samples were amended with Hg(NO_3_)_2_ to achieve final Hg concentrations ranging from 50 nM–500 μM (equivalent to 110 nmol–80 μmol Hg g^-1^ dry weight, Supplementary Table S1). It is important to note that Hg(II) was added to non-living subcellular fractions at elevated concentrations to ensure saturation of thiol binding sites, thereby allowing accurate quantification via a titration procedure. Samples were incubated for 48 hours in a N_2_-filled glovebox to ensure a chemical equilibrium condition to complete the complexation between Hg(II) and thiol groups. All operations were conducted in the glovebox, except for centrifugation, to minimize exposure to oxygen. Samples were kept in the dark (wrapped in aluminum foil) during preparation. After incubation, samples were freeze-dried, pressed into 5 mm pellets, and stored in centrifuge tubes with loosely capped lids inside the glovebox overnight. Tubes were then sealed tightly and stored at -20 °C until beamline analysis.

Details of Hg EXAFS data collection and analysis are as follows: Hg L_*III*_-edge EXAFS data were collected at 77K using a liquid nitrogen cryostat in fluorescence mode with a 64-element monolithic germanium (Ge) detector with the Xspress-4 digital pulse processor at the I20-Scanning beamline, Diamond Light Source, UK (Diaz-Moreno et al., 2018). The X-rays were generated from a wiggler insertion device, and the beamline was equipped with a four-bounce Si(111) monochromator, producing highly stable monochromatic X-rays with a final beam spot size of 400 × 300 μM (*h* × *v*) on the sample. EXAFS spectra were collected with an energy step of 0.3 eV and a constant *k*-step of 0.04 Å^-1^ in the EXAFS region, spanning from -200 to 800 eV relative to the Hg L_*III*_-edge (12.284 eV). The incident energy was calibrated using a gold foil at the Au L_*III*_-edge (11.919 eV). Two or three XAS scans were collected and averaged using Athena software (Ravel and Newville, 2005). Data normalization was conducted within the 12.200−12.600 eV range, and background removal was performed using a 6–8 knot spline function over the *k*-range of 2.7−13.5 Å^-1^ to extract EXAFS from the XAS spectra. Data reduction and Fourier-transformed *R*-space fitting were carried out using WinXAS (Ressler, 1998) and FEFF-7 (Rehr et al., 1994; Zabinsky et al., 1995).

Modeling of Hg-thiol complexes included single scattering paths for Hg-S, Hg-O/N in the first coordination shell, Hg-C and Hg-S-S (disulfide) in second shell as well as a multiple scattering (MS) path for S-Hg-S, were consistent with models previously to interpret Hg(II)-NOM complexation (Song et al., 2018). Modeling of Hg–thiol complexes included single-scattering paths for Hg–S and Hg–O/N in the first coordination shell, Hg–C and Hg–S–S (disulfide) in the second shell, as well as a multiple-scattering (MS) path for S–Hg–S, were consistent with those previously used to interpret Hg(II)–NOM complexation (Song et al., 2018). The structural model for metallic ${\text{Hg}}_{\left(l\right)}^{0}$ and metacinnabar (β-HgS) was generated in FEFF-7 using atomic coordinates from Bone et al. (2014) and Skyllberg and Drott (2010), respectively with coordination numbers fixed to theoretical values.

### 2.6 Thiol quantification

The thiol group concentration in each individual sample (*C*_*RS*_, μmol g^−1^) was calculated using finally determined Hg concentrations (by DMA) and coordination numbers (CNs) obtained from EXAFS, as described in Equation 1.


$$C_{\text{RS}}=2C_{Hg_{final}}\times \frac{{\text{CN}}_{\text{S}}/2}{{\text{CN}}_{\text{S}}/2+{\text{CN}}_{\text{O/N}}/2+{\text{CN}}_{{\text{Hg}}^{0}}/6+{\text{CN}}_{\text{HgS}}/4}$$ (1)

where *C*_*H*_*g*__*final*__ is the concentration of Hg (in μmol g^−1^), and CN_S_, CN_O/N_, ${\text{CN}}_{{\text{Hg}}^{0}}$, and CN_HgS_ are the EXAFS model fitted first-shell coordination numbers of chemical bonds Hg-S in Hg(RS)_2_, Hg-O/N in Hg(RO/N)_2_, Hg-Hg in liquid elemental Hg [${\text{Hg}}_{\left(l\right)}^{0}$] and Hg-S in metacinnabar (β-HgS) respectively, with the denominators 2, 2, 6, and 4 representing the theoretical CNs of Hg-S, Hg-O/N, Hg-Hg, and Hg-S in these chemical forms, respectively. Importantly, only data for samples in which Hg-O/N (oxygen and nitrogen bonding cannot be separated by EXAFS) were observed in the first coordination shell were used in the calculation of *C*_*RS*_. The underlying assumption is that only if the much weaker bonds to O/N atoms are detected, all thiol groups are saturated by Hg(II) and thus can be determined by Hg(II) titration (Skyllberg et al., 2006). In samples without detectable O/N involvement, the calculated thiol content is underestimated. The chemical forms ${\text{Hg}}_{\left(l\right)}^{0}$ and β-HgS are expected to form in parallel to Hg-thiol complexes. Only Hg species (Hg(RS)_2_, Hg(RO/N)_2_, ${\text{Hg}}_{\left(l\right)}^{0}$ and β-HgS) improving the merit-of-fit, ∑(model − experiment)^2^/∑experiment^2^, by more than 10% were considered significant.

It should be noted that the reported thiol concentration (*C*_RS_), derived from Equation 1, includes mass of the extraction reagents (for the cytoplasmic and periplasmic fractions) or the mass of growth medium (for the extracellular fraction). Thus, it does not represent the thiol concentration of the “pure” compartment alone. A more appropriate measure is the concentration of thiols per cell (μmol cell^-1^), where the cell densities were estimated from OD_600_. Total membrane thiols (inner + outer membranes) concentration per cell were calculated by subtracting the cytoplasmic and periplasmic thiols from that of whole-cell. Thiol concentrations express per gram of TOC (μmol g^-1^ C) were also reported after correcting for reagent-derived TOC, although this correction introduces greater uncertainty. In contrast, thiol concentrations normalized on a per-cell basis (μmol cell^-1^) are not affected by this issue and are therefore used throughout the study.

### 2.7 Statistical analysis and uncertainty estimation

Uncertainties in calculated thiol group concentrations were propagated from an estimated 10% uncertainty in EXAFS coordination numbers (CNs), along with the standard deviations of Hg concentrations (Supplementary Table S1) and cell densities (Supplementary Figure S1). Statistical differences in thiol content between samples were assessed using a Z-test as each Hg EXAFS dataset originated from a single sample. The Z-test was calculated as:


$$z=\frac{\left|\mu_{1}-\mu_{2}\right|}{\sqrt{\sigma_{1}^{2}+\sigma_{2}^{2}}}$$ (2)

where μ_1_ and μ_2_ are thiol concentrations; σ_1_ and σ_2_ are their respective propagated uncertainties. A difference was considered statistically significant at the 95% confidence level when *z* > 1.96. All calculations and statistical analyses were performed in R (Version 4.4.3) (R Core Team, 2025), with uncertainty propagation calculated by the first-order Taylor series method, as implemented in the R package errors (Ucar et al., 2018).

## 3 Results and discussion

### 3.1 Mass and TOC of subcellular fractions

Dry mass, total organic carbon (TOC), and total sulfur (TS) were quantified in extracellular and subcellular fractions at the exponential growth phase (OD_600_ ~0.5). Background contributions from media and extraction reagents were subtracted to correct the values. As shown in Supplementary Tables S2–S4, the two subcellular fractionation protocols produced consistent results, with average recoveries in the range of 80%–130%.

Averaged values from both protocols are summarized in Table 1. Specifically, the dry mass values for the extracellular, cytoplasmic, periplasmic, inner membrane, and outer membrane fractions were (6.0 ± 1.7) × 10^−12^, (1.2 ± 0.2) × 10^−12^, (5.0 ± 0.5) × 10^−13^, (2.4 ± 0.4) × 10^−13^ and (6.0 ± 2.0) × 10^−14^ g cell^−1^, respectively. The whole-cell dry mass was (1.6 ± 0.2) × 10^−12^ g cell^-1^, approximately five times greater than that of *E. coli* grown in nutrient-poor medium (2.8 × 10^−13^ g cell^-1^) (Dennis and Bremer, 1974; Neidhardt et al., 1990), likely due to bacterial species and growth condition differences. The whole-cell dry mass was 0.36 ± 0.05 g L^−1^ of bacterial culture volume, closely matching the previously reported value of 0.42 g L^−1^ for *G.sulfurreducens* (Engel et al., 2020).

**Table 1:** Concentration of dry mass, total organic carbon (TOC), total sulfur (TS), and thiol groups (RSH) across extracellular and subcellular fractions of *G. sulfurreducens* (mean ± SD or propagated error).

**Sub**	**Mass**	**TOC**	**TS**	**RSH concentration^†^**	**RSH concentration^‡^**	**RSH density (RSH/TOC)^§^**
	**(**×**10**^−13^ **g cell**^−1^**)**		**(**×**10**^−11^ ***μ*mol cell**^−1^**)**		**(μmol g^-1^ C of whole-cell)**	**(μmol g^-1^ C of each subcellular)**
Ex	60 ± 17	19 ± 9	n.d.	5.7 ± 2.8		29 ± 23
Cy	12 ± 2	4.3 ± 0.7	10.4 ± 1.9	1.5 ± 0.3	22 ± 4	36 ± 13
Pe	5 ± 0.5	2.6 ± 1.4	2.4 ± 0.4	>3.6 ± 1.1	>50 ± 16	>137 ± 87
Me	3 ± 0.4	1.8 ± 0.2	16.3 ± 1.9	8.3 ± 1.9*	116 ± 29	467 ± 153
IM	2.4 ± 0.4	1.6 ± 0.2	15.6 ± 1.9	7.1 ± 1.6^#^	100 ± 25	451 ± 158
OM	0.6 ± 0.2	0.2 ± 0.03	0.7 ± 0.1	1.2 ± 0.3^#^	17 ± 4	600 ± 299
De	0.3 ± 0.2	0.13 ± 0.04	3.0 ± 2.3	n.d.	n.d.	n.d.
WC	16 ± 2	7.2 ± 1.0	41.6 ± 6.8	13.5 ± 1.6	188 ± 30	188 ± 30

Sub, Subcellular fraction; Ex, Extracellular; Cy, Cytoplasm; Pe, Periplasm; Me, Membranes; IM, Inner membrane; OM, Outer membrane; De, Debris; WC, Whole-cell.

^†^RSH concentration expressed by per cell (μmol cell^-1^).

^‡^RSH concentration expressed per gram of whole-cell TOC (μmol g^-1^ C).

^§^“RSH density” refers to the thiol density (RSH/TOC) for each subcellular compartment.

n.d., not determined.

^*^Membrane RSH (IM + OM) estimated by subtracting cytoplasmic and periplasmic values from that of the whole-cell.

^#^IM and OM RSH estimated assuming a 6:1 ratio based on Gutensohn (2023) and Wang et al. (2016).

Errors in mass, TOC, and TS are standard deviations (SD) from triplicate measurements; RSH errors are propagated SDs from uncertainties in EXAFS CNs, Hg concentrations, and cell density.

TOC content in the extracellular, cytoplasmic, periplasmic, inner membrane, and outer membrane fraction was (1.9 ± 0.9) × 10^−12^, (4.3 ± 0.7) × 10^−13^, (2.6 ± 1.4) × 10^−13^, (1.6 ± 0.2) × 10^−13^ and (2.0 ± 0.3) × 10^−14^ g cell^-1^, respectively. A strong correlation was found between dry mass and TOC (Pearson's *r* = 0.99, *p* < 0.01; Supplementary Figure S2). Whole-cell TOC was (7.2 ± 1.0) × 10^−13^ g cell^-1^, representing (46 ± 2)% of the total dry mass, while total nitrogen (TN) accounted for (12 ± 1)%. Both values are consistent with those reported for *E.coli* (50% TOC and 14% TN) (Neidhardt et al., 1990).

When the extracellular fraction was excluded, the cytoplasm comprised 59% of whole-cell dry mass and 48% of TOC. The periplasm, inner membrane, and outer membrane contributed 25%, 12%, and 3% of dry mass, and 30%, 18%, and 2% of TOC, respectively (Table 2). These distributions are consistent with reports of Gram-negative bacteria (e.g., *E. coli, Salmonella typhimurium*), with the cytoplasmic and periplasmic spaces occupying approximately 60%–90% and 20%–40% of total cell volume, respectively (Prochnow et al., 2019; Stock et al., 1977).

**Table 2:** Distribution (%) of dry mass, total organic carbon (TOC), total sulfur (TS), and thiol groups (RSH) across extracellular and subcellular fractions of *G. sulfurreducens* (mean ± SD or propagated error).

**Sub**	**Mass**	**TOC**	**TS**	**RSH**	**Mass**	**TOC**	**RSH**
	**(Whole-cell, no extracellular component)** ^a^				**(Cell culture, with extracellular component)** ^b^		
Ex	n.a.	n.a.	n.a.	n.a.	75 ± 27	69 ± 28	30 ± 16
Cy	59 ± 13	48 ± 11	32 ± 7	11 ± 2	15 ± 4	15 ± 5	8 ± 2
Pe	25 ± 4	30 ± 17	7 ± 1.4	27+-8	6 ± 1	9 ± 6	19+-7
Me	15 ± 3	20 ± 4	51 ± 8	62 ± 16^*^	4 ± 1	6 ± 2	44 ± 14
IM	12 ± 2	18 ± 4	49 ± 8	53 ± 14^#^	3 ± 1	6 ± 1	37 ± 12
OM	3 ± 1	2 ± 0.5	2 ± 1	9 ± 2^#^	0.8 ± 0.3	0.7 ± 0.3	6 ± 2
De	1 ± 0.9	2 ± 0.5	9 ± 7	n.d.	0.4 ± 0.2	0.5 ± 0.2	n.d.
WC	100 ± 14	100 ± 21	100 ± 13	100 ± 16	25 ± 6	31 ± 8	70 ± 18

Sub, Subcellular fraction; Ex, Extracellular; Cy, Cytoplasm; Pe, Periplasm; Me, Membranes; IM, Inner membrane; OM, Outer membrane; De, Debris; WC, Whole-cell.

^a^Proportion in whole-cell, excluding extracellular component (%).

^b^Proportion in cell culture, including both whole-cell and extracellular components (%).

n.a., not available; n.d., not determined.

^*^Membrane RSH (IM + OM) estimated by subtracting cytoplasmic and periplasmic values from that of the whole-cell.

^#^IM and OM RSH estimated assuming a 6:1 ratio based on Gutensohn (2023) and Wang et al. (2016).

Errors in mass, TOC, and TS are standard deviations (SD) from triplicate measurements; RSH errors are propagated SDs from uncertainties in EXAFS CNs, Hg concentrations, and cell density.

When both extracellular and cellular components were included (Table 2), the extracellular fraction accounted for 75% of the total dry mass and 69% of TOC in the cell culture, with relatively high uncertainties (~30%) due to the correction of background contributions from the growth medium The cytoplasm, periplasm, inner membrane, and outer membrane contributed 15%, 6%, 3%, and ~1% of dry mass, and 15%, 9%, 6%, and ~1% of TOC, respectively. The debris fraction, likely composed of residual membrane material and incompletely lysed cells, contributed less than 2% to both the dry mass and TOC of the whole-cell (Supplementary Tables S2, S3).

### 3.2 Sulfur speciation

Total sulfur (TS) accounted for 0.85% of whole-cell dry mass (Supplementary Table S4), consistent with reported values of 0.5%–1% in bacterial dry mass (Kertesz, 2000). Sulfur was predominantly associated with the inner membrane (49%), followed by the cytoplasm (32%) (Table 2).

Sulfur K-edge XANES analysis (uncertainty estimated at ± 5% in the quantity of specified S forms) was performed on the extracellular, cytoplasmic, periplasmic, and whole-cell fractions. Because the energy separation between disulfide (RSSR), monosulfide (RSR), and thiol (RSH) is very small (< 1 eV), these species cannot be individually resolved in S K-edge XANES. However, their combined signal is readily distinguishable from other sulfur oxidation states and can be quantified with confidence. For this reason, the sum of these species is reported as reduced organic sulfur (Org-S_*RED*_). In other words, S XANES provides only a semi-quantitative estimate of thiols through the Org-S_*RED*_ fraction.

**Figure 2:**
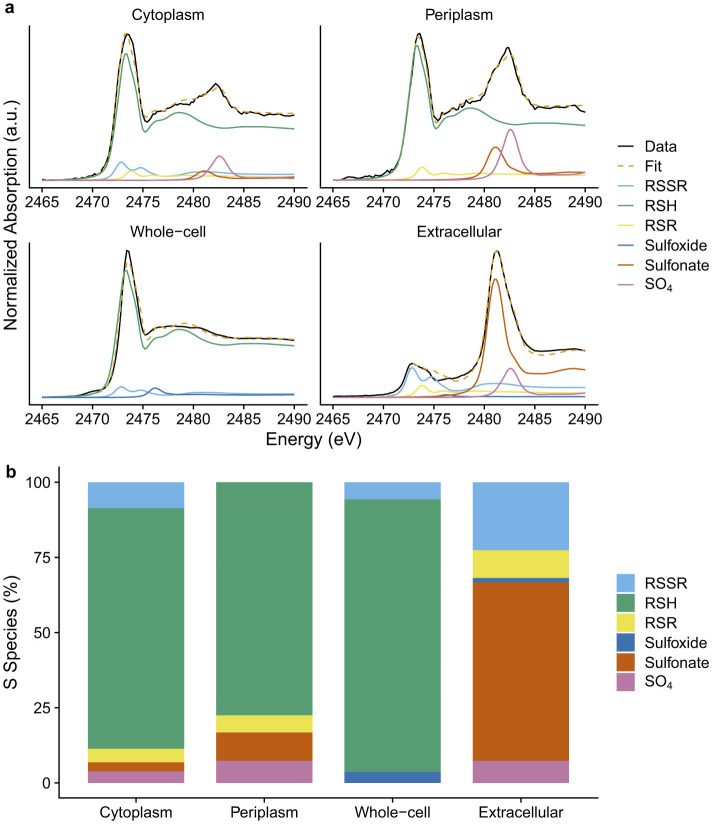
**(a)** Sulfur K-edge XANES spectra and linear combination fitting (LCF) and **(b)** sulfur speciation (% of total sulfur) in cytoplasmic, periplasmic, whole-cell, and extracellular fractions of *G. sulfurreducens*.

Org-S_*RED*_ accounted for ~90% of total sulfur in the cytoplasmic, periplasmic, and whole-cell samples, but accounted only for ~30% in the extracellular fraction (Table 3 and Figure 2). These findings are consistent with our previous study employing physical cell lysis (i.e., freeze-thaw and ultrasonication), in which Org-S_*RED*_ accounted for approximately 90% of total sulfur in membrane and whole-cell samples, and around 25% in extracellular components of *G. sulfurreducens* (Song et al., 2020). This suggests that the subcellular fractionation method used in the present study does not alter sulfur speciation or subcellular thiols.

**Table 3:** Sulfur speciation (%) in subcellular fractions of *G. sulfurreducens* determined by sulfur K-edge XANES.

**Species (%)**	**Cytoplasm**	**Periplasm**	**Whole-cell**	**Extracellular**
RSSR	9	0	6	23
RSH	80	78	91	0
RSR	5	6	0	9
Sulfoxide	0	0	4	1
Sulfonate	3	9	0	59
SO_4_	4	7	0	7
**Org-S**_RED_^*^	**94**	**84**	**97**	**32**

^*^Org-S_*RED*_ represents reduced organic sulfur, including thiol (RSH), monosulfide (RSR), and disulfide (RSSR). The uncertainty of the reported values is approximately 5%.

### 3.3 Chemical speciation of mercury characterized by Hg L_*III*_-edge EXAFS

#### 3.3.1 Hg(II)-thiol coordination

**Figure 3:**
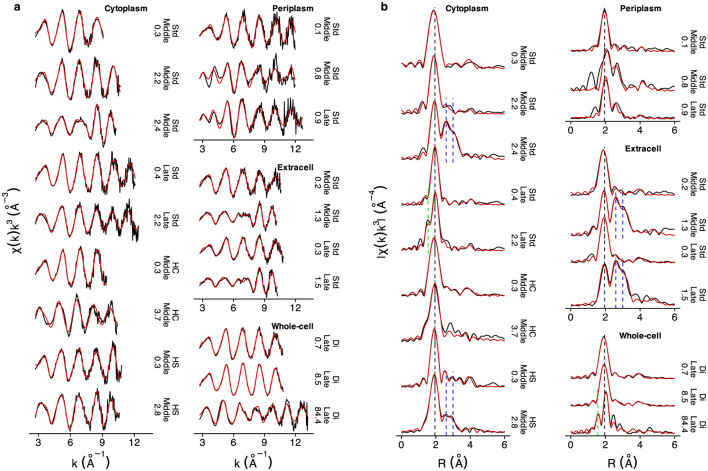
Hg L_*III*_-edge EXAFS spectra (black solid lines) and corresponding model fits (red solid lines) collected at 77 K for cytoplasmic, periplasmic, extracellular, and whole-cell samples: **(a)** EXAFS spectra in *k*-space and **(b)** Fourier-transformed (FT) spectra (not phase-corrected). The vertical black dashed line represents the Hg-S bond at 2.35 Å, the green dashed line represents the Hg-O bond at 2.05 Å, and the two blue dashed lines represent the Hg-Hg_1_ and Hg-Hg_2_ bond of liquid ${\text{Hg}}_{\left(l\right)}^{0}$ at 2.99 Å and 3.46 Å, respectively. The annotations to the right of each spectrum indicate the treatment, growth phase, and final Hg concentration (in μmol g^-1^).

The interaction between Hg(II) and thiol groups (RSH) was confirmed for all samples by the important contribution from first-shell Hg-S scattering path in all EXAFS spectra (Figure 3 and Table 4). At relatively low Hg concentrations (e.g., 0.1–0.9 μmol g^−1^ in periplasmic/extracellular samples, 0.3–0.4 μmol g^−1^ in cytoplasmic samples, and 0.7–8.5 μmol g^−1^ in whole-cell samples), EXAFS spectra showed only Hg-S coordination, with an average bond distance of 2.35 Å (ranging from 2.34 to 2.36 Å), as reflected by the FT peak at 1.9A in *R*-space, uncorrected for phase shift (Figure 3b and Table 4). A weak feature near 4.63 Å (4 Å in *R*-space) reflects the characteristic four-legged Hg-S-Hg-S multiple-scattering path. Together, these features are consistent with a linear, two-coordinated Hg(RS)_2_ structure (bond angle 180°). These observations align with previous studies on Hg-thiol interactions in low molecular mass thiols (Jalilehvand et al., 2013, 2006; Manceau and Nagy, 2008), natural organic matter (NOM) (Skyllberg et al., 2006; Song et al., 2018), and *G. sulfurreducens* membranes (Mishra et al., 2017; Song et al., 2020).

**Table 4:** First coordination shell model fits to full *k*-space Hg L_*III*_-edge EXAFS data for cytoplasmic, periplasmic, extracellular, and whole-cell samples added different concentrations of Hg(II).

**Sample**	**Hg_*final*_**	**ΔE_0_**	**Hg-Hg at Hg(RS)**_**2**_			**Hg-O/N at Hg(RO/N)**_**2**_			**Hg-Hg at ** ${\text{Hg}}_{\left(l\right)}^{0}$			**Hg-S at HgS**			**Hg species (%)**				*C*_RS_^*^	**RS^#^**
			**CN**	**R**	σ^2^	**CN**	**R**	σ^2^	**CN**	**R**	σ^2^	**CN**	**R**	σ^2^	**Hg(RS)**_2_	**Hg(RO/N)**_2_	** ${\text{Hg}}_{\left(l\right)}^{0}$ **	**HgS**		
Cy-Mid-Std	0.3	5.6	1.62	2.35	0.006										100	0	0	0		
Cy-Mid-Std	2.2	4.3	0.65	2.35	0.003	0.12	2.05	0.003							85	15	0	0	3.8 ± 0.3	15.5 ± 2.7
Cy-Mid-Std	2.4	5.6	0.76	2.35	0.003				1.02	2.99	0				69	0	31	0	>3.3 ± 0.4	>6.4 ± 1.1
Cy-Late-Std	0.4	8.7	2.1	2.35	0.004										100	0	0	0		
Cy-Late-Std	2.2	4.9	0.71	2.37	0.003	0.31	2.05	0.003							69	31	0	0	3.0 ± 0.2	5.7 ± 0.5
Cy-Mid-HC	0.3	7.7	1.97	2.34	0.004										100	0	0	0		
Cy-Mid-HC	3.7	4.4	0.59	2.41	0.003	0.28	2.11	0.003				0.15	2.53	0.005	62	30	0	8	4.6 ± 0.7	13.7 ± 2.6
Cy-Mid-HS	0.3	3.8	1.5	2.36	0.004										100	0	0	0		
Cy-Mid-HS	2.8	7.7	0.57	2.38	0.003	0.36	2.11	0.004	0.27	2.99	0.003				56	35	9	0	3.1 ± 0.3	11.7 ± 1.5
Pe-Mid-Std	0.1	9.9	1.77	2.35	0.003										100	0	0	0		
Pe-Mid-Std	0.8	11	0.53	2.37	0.003							0.16	2.53	0.015	87	0	0	13	>1.4 ± 0.4	>36 ± 11
Pe-Late-Std	0.9	11	0.91	2.36	0.003										100	0	0	0	>1.7 ± 0.4	>20 ± 4
Ex-Mid-Std	0.2	6.7	1.88	2.34	0.008										100	0	0	0		
Ex-Mid-Std	1.3	7	0.65	2.36	0.003	0.46	2.06	0.015	1.38	2.99	0.003				41	29	29	0	1.1 ± 0.3	57 ± 28
Ex-Late-Std	0.3	9.1	1.85	2.35	0.003										100	0	0	0		
Ex-Late-Std	1.5	6.8	0.89	2.36	0.004				2.01	2.99	0.003				57	0	43	0	1.8 ± 0.4	53 ± 14
WC-Late-Di	0.7	5.9	2.08	2.35	0.005										100	0	0	0		
WC-Late-Di	8.5	8.1	2.18	2.34	0.004										100	0	0	0		
WC-Late-Di	84.4	6.6	0.67	2.42	0.003	0.3	2.05	0.003	0.88	2.99	0.015				53	24	23	0	89.5 ± 7.0	135 ± 16
IM-Mid-Std^†^																				71 ± 16
OM-Mid-Std^†^																				12 ± 3

Sample names follow the format “Subcellular Component-Growth Phase-Treatment,” where:

Cy, Cytoplasm; Pe, Periplasm; Ex, Extracellular; Pe, Periplasm; WC, Whole-cell; IM, Inner membrane; OM, Outer membrane.

“Mid” refers to the middle-exponential phase (2 days), and “Late” refers to the late-exponential phase (3 days).

“Std” = Standard growth medium, “HC” = High-carbon medium (3 × fumarate), “HS” = High-sulfur medium (10 × sulfate), “Di” = Cell disruptor treatment.

OD_600_ represents the optical density measured at 600 nm.

Hg_*add*_ indicates the initial Hg(II) concentration added (μmol g^-1^), while Hg_*final*_ represents the final total Hg concentration determined after EXAFS experiments (μmol g^-1^).

^*^ denotes the thiol concentration calculated from the EXAFS-derived CN and Hg_*final*_ using Equation 1 (± propagated error). This value reflects thiols in the extracted samples together with reagents or medium and therefore does not represent the “pure” compartment alone.

^#^ indicates thiol concentration per cell (× 10^−12^ μmol cell^-1^) ± propagated error.

^†^ Calculated using an inner-to-outer membrane ratio of 6:1, based on Gutensohn (2023) and Wang et al. (2016).

*EXAFS Fitting Parameters and Uncertainties:*

Coordination Number (CN): 10%;

Bond Distance (R): 0.01–0.02 Å;

Edge Energy Shift (ΔE_0_): 1–3 eV;

Debye–Waller Factor (σ^2^): constrained between 0.003–0.015 Å^2^;

Amplitude Reduction Factor ($S_{0}^{2}$): fixed at 0.9 for all samples.

A second Hg-C shell was also identified, with an average bond distance of 3.34 Å, well in agreement with the Hg(RS)_2_ structure (Skyllberg et al., 2006; Song et al., 2018). EXAFS fitting employed correlated coordination numbers (CN) of 1:1:1 for Hg-S, Hg-C, and MS paths to maintain consistency of the Hg(RS)_2_ structure (Supplementary Table S5).

#### 3.3.2 Hg-O/N coordination

At higher Hg concentrations, first-shell Hg-O/N bonds were identified (representing Hg-O bonds with carboxyl/phenol groups and/or possibly Hg-N bonds with amino groups, indistinguishable by EXAFS). The average Hg-O/N bond distance was 2.07 Å, ranging from 2.05 to 2.11 Å (as denoted by the vertical green dashed line in Figure 3), indicative of Hg(RO/N)_2_ structures, consistent with previous studies (Skyllberg et al., 2006; Song et al., 2018, 2020).

#### 3.3.3 Liquid elemental Hg [${\text{Hg}}_{\left(l\right)}^{0}$]

**Figure 4:**
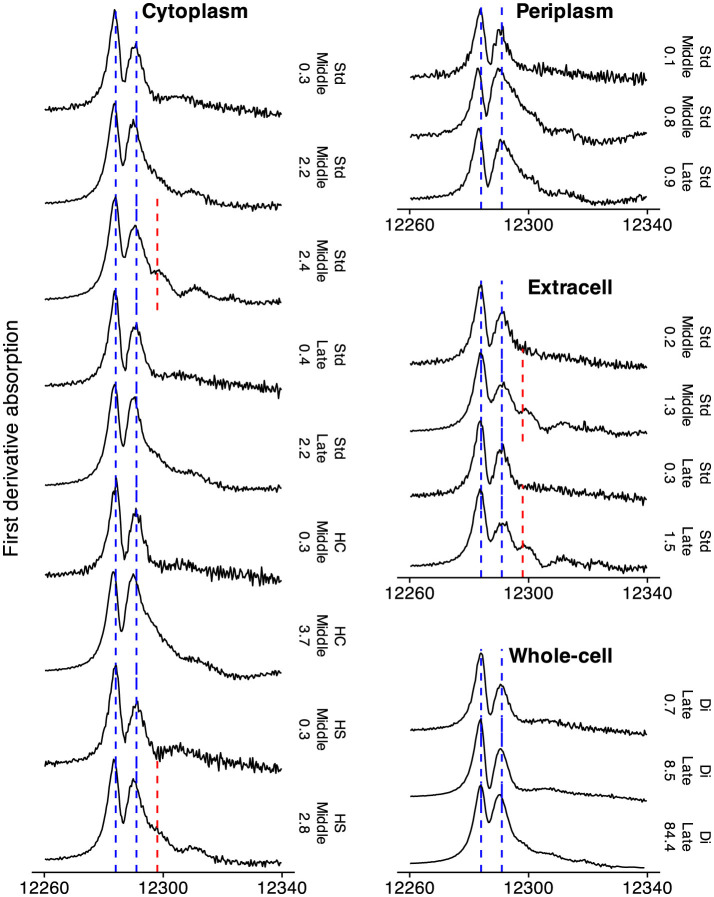
First-derivative Hg L_*III*_-edge XANES spectra (black solid lines) collected at 77 K for cytoplasmic, periplasmic, extracellular, and whole-cell samples. Vertical blue dashed lines indicate characteristic Hg(II) features at 12.284 and 12.291 eV, while the red dashed line marks the liquid ${\text{Hg}}_{\left(l\right)}^{0}$ feature at 12.298 eV. Annotations to the right of each spectrum denote the treatment, growth phase, and final Hg concentration (μmol g^−1^).

${\text{Hg}}_{\left(l\right)}^{0}$ was detected in cytoplasmic, extracellular, and whole-cell samples exposed to relatively higher Hg concentrations (2.4–2.8 μmol g^−1^, 1.3–1.5 μmol g^−1^, and 84.4 μmol g^−1^, respectively, as denoted by the blue dashed lines in Figure 3b). The *R*-space spectra exhibited a distinct peak at 2.6 Å and a shoulder at 3.0 Å (uncorrected for phase shift), corresponding to Hg-Hg_1_ and Hg-Hg_2_ EXAFS scattering paths at 2.99 and 3.46 Å, respectively. These characteristics were further supported by the *k*-space spectra, which displayed dampened oscillations below 8 Å^-1^, smoother peak shapes, and reduced amplitudes, features typical of ${\text{Hg}}_{\left(l\right)}^{0}$ and α-${\text{Hg}}_{\left(s\right)}^{0}$ (the latter observed below the freezing point of ${\text{Hg}}_{\left(l\right)}^{0}$, -38.83 °C) (Jew et al., 2011). The presence of ${\text{Hg}}_{\left(l\right)}^{0}$ was further confirmed by first-derivative XANES analysis, which showed peaks at 12.284 and 12.291 eV and a dip at 12.287 eV, along with an additional shoulder at 12.298 eV (Figure 4), consistent with previous reports (Bone et al., 2014; Mishra et al., 2011).

The formation of ${\text{Hg}}_{\left(l\right)}^{0}$ likely occurs through initial reduction of Hg(II) to ${\text{Hg}}_{\left(aq\right)}^{0}$, followed by aggregation when (local) ${\text{Hg}}_{\left(aq\right)}^{0}$ concentrations become sufficiently high, as covered by the following reactions (Morel and Hering, 1993):

Hg^2^+ + 2 e^-^ <=> ${\text{Hg}}_{\left(aq\right)}^{0}$, log *K* = 22.32;

${\text{Hg}}_{\left(aq\right)}^{0}$ <=> ${\text{Hg}}_{\left(g\right)}^{0}$, log *K* = 2.59;

${\text{Hg}}_{\left(aq\right)}^{0}$ <=> ${\text{Hg}}_{\left(l\right)}^{0}$, log *K* = 6.48.

As for sample preparation, freeze-drying was performed at approximately -50 °C in this study, below the melting point of ${\text{Hg}}_{\left(l\right)}^{0}$ (-38.8 °C). Parallel experiments in which *G. sulfurreducens* cells or spheroplasts were exposed to Hg(II) for 1 h and then either frozen or freeze-dried showed Hg losses of 9%–19% in frozen samples and 12%–22% in freeze-dried samples, indicating only minimal additional volatilization during the process of freeze-drying (as compared to frozen conditions) (Gutensohn et al., unpublished data).

#### 3.3.4 Potential Hg-disulfide (RSSR) coordination

At low Hg concentration levels (0.1 μmol g^−1^), a linear Hg(RS)_2_ structure dominated the periplasmic samples, similar to other fractions. However, at higher Hg concentrations (0.8 and 0.9 μmol g^−1^), distinct spectral features appeared. Specifically, the *k*-space spectra displayed three oscillation cycles with well-defined peaks in the 2.7–6 Å^-1^ region, in contrast to the two oscillation cycles observed in all other samples (Figure 3a). This unusual spectral pattern, not previously documented in the literature, could only be modeled by incorporating a significant contribution from second-shell sulfur (possibly RSSR, disulfide) interactions at 2.85 Å, with high CNs (2.0 at 0.8 μmol g^−1^ and 2.6 at 0.9 μmol g^−1^, Supplementary Table S5).

#### 3.3.5 Metacinnabar (β-HgS)

Metacinnabar (β-HgS) contributed to the EXAFS spectrum of the 0.8 μmol g^−1^ periplasmic sample, accounting for 13% of total Hg species. This is species yields a shift in the first *R*-space peak to a slightly longer distance (2.1 Å vs. 1.9 Å, Figure 3b), reflecting the longer Hg-S bond length in the tetrahedral β-HgS structure (2.53 Å) compared to the linear Hg(RS)_2_ complex (2.35 Å). Notably, β-HgS demonstrates a second shell Hg-Hg interaction at 4A in the uncorrected Fourier transform (Supplementary Table S5) which overlaps with the four-legged multiple scattering path Hg-S-Hg-S-Hg, characteristic of the linear S-Hg-S geometry in Hg(RS)_2_. In contrast, Hg(RS)_3_ and Hg(RS)_4_ structures lack multiple scattering features near this region in *R*-space. Although β-HgS (8%) could be fitted in the high-carbon cytoplasmic sample, it remains inconclusive given the 10% model threshold.

No significant Hg-Cl coordination was detected by Hg L_*III*_-edge EXAFS nor in the XANES region, despite presence of 6.5 mmol L^-1^ Cl- in the extracellular fraction. PHREEQC calculations indicate that Hg(II) is dominated by Hg(RS)_2_ (as well as Hg(OH)_2_ at higher Hg loadings around pH 7), while Hg-Cl species account for far less than 1% of total Hg and are therefore thermodynamically negligible.

### 3.4 Subcellular thiols concentrations

#### 3.4.1 Extracellular, cytoplasmic, periplasmic, and whole-cell thiols

Calculated thiol concentrations in each sample (*C*_RS_, uncorrected for effect from reagents or culture medium, see Section 2.6 above for details), and the corresponding per cell thiol concentrations (μmol cell^-1^) are presented in Table 4. In the cytoplasm, thiol concentration was (1.5 ± 0.3) × 10^−11^ μmol cell^-1^ in the middle-exponential phase (OD_600_ ~0.51) and significantly decreased to (5.7 ± 0.5) × 10^−12^ μmol cell^-1^ (*z* > 1.96) in the late-exponential phase (OD_600_ ~0.83). Extracellular thiol concentrations were (5.7 ± 2.8) × 10^−11^ and (5.3 ± 1.4) × 10^−11^ μmol cell^-1^ in the middle- and late- exponential phases, respectively, with no significant difference observed (*z* < 1.96). Periplasmic thiol levels could not be directly determined due to incomplete thiol saturation by Hg(II), as indicated by the absence of Hg-O/N coordination in EXAFS model fits. Therefore, EXAFS results provide only lower-bound estimates of thiol concentrations: (3.6 ± 1.1) × 10^−11^ and (2.0 ± 0.4) × 10^−11^ μmol cell^-1^ in the middle- and late-exponential phases, respectively.

Thiols concentrations (in μmol cell^-1^) for *G. sulfurreducens* (as well as *P. mercurii* ND132), determined in this and previous studies using various cell isolation and analytical methods, are summarized in Table 5. Gutensohn et al. (2023a) reported extracellular LMM-RSH concentrations of 3.6 × 10^−12^ μmol cell^-1^ during the middle exponential phase and 1.5 × 10^−12^ μmol cell^-1^ during the late exponential phase, approximately one order of magnitude lower than ours (5 × 10^−11^ μmol cell^-1^). This may be due to the LC-ESIMS/MS method that enabled quantification of only (eight) specific LMM-RSH compounds, whereas our EXAFS approach captures all thiol groups capable of coordinating with Hg. Adediran et al. (2019) reported concentrations of 1 × 10^−12^ μmol cell^-1^ at 6 and 48 hours, which are roughly 50-fold lower than our values, likely due to their use of an assay buffer with 20-fold lower carbon source than the standard growth medium used in this study (Table 5).

**Table 5:** Extracellular and subcellular thiol concentrations in *Geobacter sulfurreducens* PCA and *Pseudodesulfovibrio mercurii* ND132 during middle and late exponential growth phases, determined using different cell isolation and analytical methods.

**Bacterium**	**Concentration (μmol cell^-1^)**	**Phase**	**Compartment**	**Separation method**	**Analytical method**	**Medium^†^**	**Reference**
**PCA**	5.7 × 10^-11^	Middle	Extracellular	0.2 μM filtration	Hg L_*III*_-edge EXAFS	Growth medium	This study
	5.3 × 10^-11^	Late					
	1.5 × 10^-11^	Middle	Cytoplasm	Spheroplasts sonication (Figure 1)			
	5.7 × 10^-12^	Late					
	>3.6 × 10^-11^	Middle	Periplasm	EDTA, sucrose, lysozyme (Figure 1)			
	>2 × 10^-11^	Late					
	1.35 × 10^-10^	Late	Whole-cell	Cell disruptor			
	8.3 × 10^-11^	Middle	Membranes^‡^	Whole-cell − cytoplasm − periplasm			
	7.1 × 10^-11^		Inner membrane	6/7 Membranes^#^			
	1.2 × 10^-11^		Outer membrane	1/7 Membranes^#^			
	3.6 × 10^-12^^c^	Middle	Extracellular	0.2 μm filtration	LC-ESIMS/MS	Growth medium	Gutensohn et al. (2023a)
	1.5 × 10^-12^^c^	Late					
	0.5 × 10^-12^^c^	Middle	Intracellular	80c MQ water, 0.2mm filtration			
	0.1 × 10^-12^^c^	Late					
	1.0 × 10^-12^^a^ (6 h)	Middle	Extracellular	0.2 μm filtration	LC-ESIMS/MS^*^	Assay buffer	Adediran et al. (2019)
	1.2 × 10^-12^^a^ (48 h)						
	7.6 × 10^-13^^b^ (6 h)	Middle	Cellular	Whole-cell − extracellular			
	4.0 × 10^-13^^b^ (24 h)						
	2.4 × 10^-13^^b^ (48 h)						
	3.8 × 10^-11^	Middle	Membranes^‡^	Ultrasonic, ultracentrifugation	Hg L_*III*_-edge EXAFS	Growth medium	Song et al. (2020)
	7.1 × 10^-11^	Late	Inner membrane (spheroplast)	Sucrose wash	Hg L_*III*_-edge EXAFS	Assay buffer	Gutensohn (2023)
	1.1 × 10^-11^	Late	Outer membrane (cell surface)	Cell wash			
**ND132**	1.6 × 10^-11^^d^	Middle	Spheroplast lysate^$^	EDTA–sucrose–lysozyme	Fluorescence (TEP-4)	MOPS buffer	Wang et al. (2016)
	2.7 × 10^-12^^d^		Outer membrane (cell wall)	Ultracentrifugation			
	1.9 × 10^-11^^e^		Whole-cell	Cell wash			

^a^ Recalculated as μmol cell^-1^ based on a cell density of 1 × 10^8^ cells mL^-1^, as reported by Adediran et al. (2019).

^b^ Concentrations of total LMM-RSH compounds were estimated as μmol cell^-1^ using cysteine data from the same study (Adediran et al., 2019).

^c^ For the middle and late exponential phases, LMM-RSH concentrations were recalculated as average values over the 0–36 h and 48–72 h intervals, respectively, based on the Figure 2 of Gutensohn et al. (2023a).

^d^ Recalculated to μmol cell^-1^ based on a cell density of 7.4 × 10^8^ cells mL^-1^ (Wang et al., 2016).

^e^ Recalculated to μmol cell^-1^ based on a cell density of 7.7 × 10^8^ cells mL^-1^ (Wang et al., 2016).

^†^ The compositions of the “Growth medium” and “Assay buffer” are provided in the SI text. The “MOPS buffer” refers to a 0.5 mM hypotonic MOPS buffer solution.

^‡^ The mixture of inner and outer membranes.

^#^ Calculated using an inner-to-outer membrane ratio of 6:1, based on Gutensohn (2023) and Wang et al. (2016).

^*^The LC-ESIMS/MS method quantified eight specific low-molecular-mass thiol compounds (LMM-RSH).

^$^Measured in 0.5 mM hypotonic MOPS buffer, which likely lysed spheroplasts and released inner membrane and cytoplasmic thiols.

Gutensohn et al. (2023a) reported intracellular LMM-RSH concentrations of 1 × 10^−13^ −5 × 10^−13^ μmol cell^-1^ (Table 5). These values represent the sum of eight targeted LMM-RSH species extracted from the cytoplasm and periplasm. Their extraction method (physical lysis by heating in 80 °C Milli-Q water for 10 min followed by 0.2 μM filtration) may not fully disrupt cellular membranes. Consequently, their “intracellular” LMM-RSH probably reflects only cytoplasmic and periplasmic thiols, excluding membranes thiols (Song et al., 2020). The reported values are therefore roughly two orders of magnitude lower than the cytoplasmic and periplasmic thiol levels quantified in our study (Table 5).

For bacteria grown in high-carbon and high-sulfate media, cytoplasmic thiol concentrations were (1.4 ± 0.3) × 10^−11^ and (1.2 ± 0.2) × 10^−11^ μmol cell^-1^, respectively, which were not significantly different from the standard growth condition (*z* < 1.96). Yu and Fein (2017) reported that an increased glucose concentration from 4 to 50 gL^−1^ increased thiol levels in the cell envelopes of both Gram-positive (e.g., *Bacillus subtilis*) and Gram-negative (*S. oneidensis*) bacteria grown in M9 minimal salt medium In nutrient-rich tryptic soy broth (TSB), however, this effect was only observed in Gram-positive *Bacillus* species. In contrast, thiol levels in Gram-negative *S. oneidensis* and *P. putida* were unaffected. Thus, the absence of increased cytoplasmic thiol levels under high-carbon conditions in our study likely reflects the already nutrient-rich standard medium (40mM fumarate and 1 gL^−1^ yeast extract). The absence of a thiol response to elevated sulfate (Na_2_SO_4_) requires further investigation.

#### 3.4.2 Inner and outer membrane thiols estimation

The total membranes (inner + outer) thiol concentration was estimated to (8.3 ± 1.9) × 10^−11^ μmol cell^-1^ by subtracting cytoplasmic and periplasmic thiols from the whole-cell value, which is in agreement with previous study reported by Gutensohn (2023). In their study, thiols on the outer and inner membranes were quantified by titrating intact cells or spheroplasts (outer membrane removed) with Hg(NO_3_)_2_ for 10 minutes, followed by Hg L_*III*_-edge EXAFS analysis. They reported a total membranes thiol concentration of 8.2 × 10^−11^ μmol cell^-1^, comprising 7.1 × 10^−11^ μmol cell^-1^ in the inner membrane and 1.1 × 10^−11^ μmol cell^-1^ in the outer membrane (inner:outer ratio of 6:1; Table 5).

Our values are roughly twice of those previously reported by Song et al. (2020) (3.8 × 10^−11^ μmol cell^-1^, Table 5), yet still comparable given biological and methodological differences. Their extraction approach relied on freeze-thaw cycles and ultrasonication to preserve membrane integrity, whereas our use of a cell disruptor likely resulted in more complete lysis and greater exposure of membranes thiols.

Wang et al. (2016) reported thiol concentrations of 1.6 × 10^−11^ and 2.7 × 10^−12^ μmol cell^-1^ in spheroplast lysate and outer membrane (cell wall), respectively, in *P. mercurii* using a fluorescence-based method, corresponding to a 6:1 ratio(Table 5). The spheroplast lysate was prepared using 0.5 mM hypotonic MOPS buffer, which likely caused lysis of the spheroplasts and released both cytoplasmic and inner membrane thiols. Given that cytoplasmic thiols comprise only a small proportion relative to inner membrane thiols, the lysate measurement predominantly reflects inner membrane thiols, if judged by the data from our study and that of Gutensohn (2023; 2023a) (Table 5).

In conclusion, both Gutensohn (2023) and Wang et al. (2016) inferred a similar inner-to-outer membrane thiol ratio of approximately 6:1. Applying this 6:1 ratio to our measured total membranes thiol concentration yields estimated values of (7.1 ± 1.6) × 10^−11^ and (1.2 ± 0.3) × 10^−11^ μmol cell^-1^ for the inner and outer membranes, respectively. Using these results, a strong positive correlation between thiol concentrations and total sulfur (TS) concentrations across all subcellular fractions was obtained, (Pearson's *r* = 0.94, *p* < 0.05; Supplementary Figure S2), which corroborates the assumption of a 6:1 ratio of thiols between the inner and outer membranes in our bacteria samples.

### 3.5 Subcellular thiols distribution

**Figure 5:**
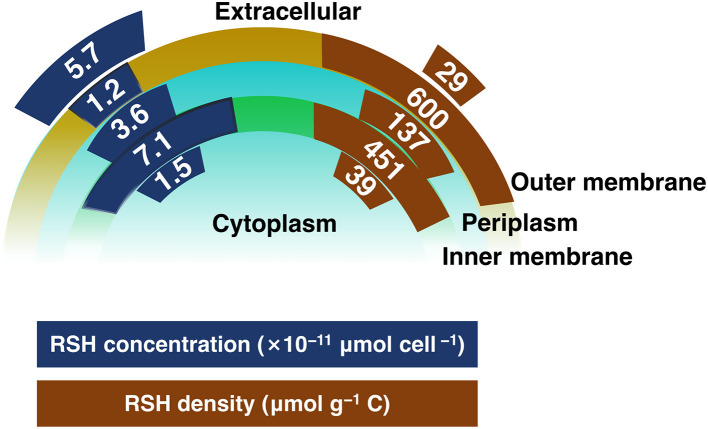
Subcellular thiol distributions in middle exponential growth stage of *G. sulfurreducens*, shown as RSH concentration per cell (×10^-11^ μmol cell^-1^; blue) and TOC-normalized thiol densities in each compartment (RSH/TOC, μmol g^-1^ C; brown).

Subcellular thiol distributions in the middle exponential-phase of *G. sulfurreducens* are summarized in Figure 5 and Table 1, expressed as RSH per cell (μmol cell^-1^). Extracellular thiols measured (5.7 ± 2.8) × 10^−11^ μmol cell^-1^. Whole-cell thiols totaled (1.35 ± 0.16) × 10^−10^ μmol cell^-1^, with membranes constituting the dominant pool, (8.3 ± 1.9) × 10^−11^ μmol cell^-1^, partitioned between the inner membrane (7.1 ± 1.6) × 10^−11^ μmol cell^-1^ and outer membrane (1.2 ± 0.3) × 10^−11^ μmol cell^-1^. Concentrations in the cytoplasmic and periplasmic fractions were (1.5 ± 0.3) × 10^−11^ and (3.6 ± 1.1) × 10^−11^ μmol cell^-1^, respectively.

The percentage distribution of thiols is shown in Table 2. When extracellular thiols are excluded, membranes account for 62 ± 16% of cellular thiols, with 53 ± 14% from the inner membrane and 9 ± 2% from the outer membrane; the cytoplasmic and periplasmic fractions contribute 11 ± 2% and 27 ± 8%, respectively. When extracellular thiols are included, they constitute 30 ± 16% of the total; membranes thiols contribute 44 ± 14%, partitioned into 37 ± 12% in the inner membrane and 6 ± 2% in the outer membrane; cytoplasmic and periplasmic thiols contribute 8 ± 2% and 19 ± 7%, respectively.

### 3.6 Hg(II) reduction and loss in subcellular fractions

**Figure 6:**
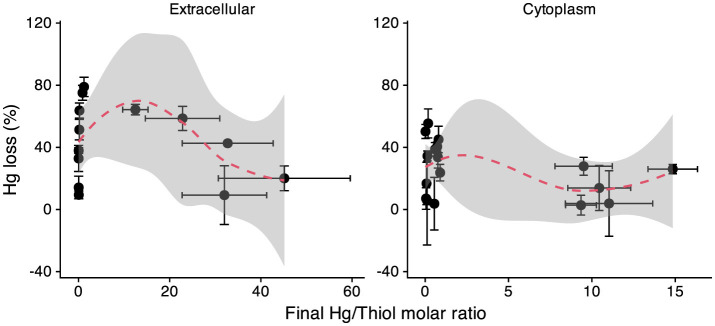
Association between Hg(II) loss (%) and the Hg-to-thiol molar ratio in the extracellular and cytoplasmic samples. Dashed lines represent loess regression curves (span = 0.9), with shaded gray areas indicating 95% confidence intervals. Error bars represent propagated standard deviations (*n* = 3–10).

It should be noted that after 48 h of reaction between Hg(II) and subcellular fractions, substantial losses (0%–79%) of total Hg were observed across subcellular fractions, including extracellular, cytoplasmic, periplasmic, and whole-cell samples (Supplementary Table S1, Figure 6). This observation is consistent with previous studies reporting Hg losses of 10%–64% in membranes (a mixture of inner and outer membranes) (Song et al., 2020), 10%–52% in separately isolated inner or outer membranes (Gutensohn, 2023), and ~56% in cells incubation (Zhang et al., 2023). Mercury loss (%) was correlated with the Hg-to-thiol molar ratio in both extracellular and cytoplasmic fractions (data for the periplasmic and whole-cell fractions were insufficient for statistical analysis). In both cases, Hg loss increased with rising Hg-to-thiol ratios, reaching a maximum at approximately 10 for the extracellular and 4 for the cytoplasmic fractions, then declined slightly (Figure 6).

This loss can be attributed to the reduction of Hg(II) to ${\text{Hg}}_{\left(g\right)}^{0}$, as evidenced by the detection of liquid ${\text{Hg}}_{\left(l\right)}^{0}$ by Hg EXAFS (see Section 3.3.3). The observed Hg losses pattern agrees with previous studies demonstrating that Hg(II) reduction by natural organic matter and by *G. sulfurreducens* membranes is promoted at higher Hg-to-thiol ratios (Jiang et al., 2015; Miller et al., 2009; Song et al., 2020). This pattern can be explained by changes in both Hg speciation and the availability of electron donors. At low Hg concentrations, electron donors from cellular components are sufficient, and the extent of Hg(II) reduction is mainly governed by Hg-ligand speciation. As the Hg-to-thiol ratio increases, Hg shifts from Hg-thiolate complexes to more reducible Hg-RO/N species, e.g., Jiang et al. (2015), enhancing reduction. However, at very high Hg loadings, electron donors become limiting, constraining the overall reduction capacity. Consequently, further increases in Hg lead to a lower percentage of Hg loss.

## 4 Implications of subcellular thiols in Hg(II) uptake

This study provides the first quantitative assessment of the subcellular distributions of biomass, total organic carbon (TOC), total sulfur (TS), and, importantly, thiols in *G. sulfurreducens*. Because Hg(II) binds strongly and preferentially to thiol groups, the abundance and organization of thiols within each compartment have substantial potential to govern Hg(II) speciation at environmentally relevant (picomolar) concentrations. These data provide essential constraints for understanding the molecular mechanisms governing Hg(II) surface interactions, cellular uptake, and subsequent transformation. Hg(II) translocation across the cell membranes is likely governed not only by the overall abundance of thiols in each compartment (e.g., thiol concentration per cell or percent distribution) but also by the local thiol density, as represented by the TOC-normalized thiol density (RSH/TOC) (Figure 5 and Table 1). The implications of compartment-specific thiols for Hg(II) speciation and cellular uptake are discussed below.

### 4.1 Extracellular thiols

The uptake of Hg(II) is highly dependent on its extracellular chemical speciation. Small neutral species such as $\text{HgC}{\text{l}}_{2_{\left(aq\right)}^{0}}$, $\text{Hg}{\left(\text{OH}\right)}_{2_{\left(aq\right)}^{0}}$, $\text{Hg}{\left(\text{SH}\right)}_{2_{\left(aq\right)}^{0}}$ (Gutknecht, 1981; Mason et al., 1996; Benoit et al., 1999a,b, 2001; Hsu-Kim et al., 2013; Zhou et al., 2017), and HgS nano-particles (Deonarine and Hsu-Kim, 2009; Zhang et al., 2012; Guo et al., 2023), are thought to cross cell membranes via passive diffusion, whereas charged or larger complexes like Hg(Cys)_2_ are proposed to be taken up via active transport (Schaefer and Morel, 2009; Schaefer et al., 2011).

In this study, the extracellular thiol concentration is approximately 6 × 10^−11^ μmol cell^-1^, representing about 30% of the total thiols in the bacteria culture. Although the TOC-normalized extracellular RSH density is relatively low (which is strongly dependent on growth conditions), extracellular thiols can still substantially influence Hg(II) speciation by the form of Hg(RS)_2_ or mixed-ligand complexes such as Hg(RS)Cl_*n*_(*OH*)_1−*n*_ in the extracellular medium Previous work in similar culture systems has shown that extracellular thiols, together with outer-membrane thiols, can modify Hg(II) speciation by promoting the formation of ternary complexes such as Hg(RS)(Mem-RS) (Adediran et al., 2019; Song et al., 2020), thereby influencing the complexes presented to the cell surface and potentially the uptake pathway.

### 4.2 Outer membrane thiols

The outer membrane has approximately 1 × 10^−11^ μmol cell^-1^ of thiols, corresponding to 6% of total thiols in the cell culture (or 10% of whole-cell thiols when the extracellular fraction is excluded). Although the absolute amount is relatively small, the outer membrane exhibited the highest thiol density (600 μmol g^-1^ C), consistent with earlier work identifying this compartment as a major site of Hg(II) retention. However, its influence on Hg(II) uptake remains debated.

One study has reported that there was no significant change in Hg(II) uptake after blocking surface thiols with the bromine-containing probe qBBr (Thomas et al., 2020). In contrast, other work suggests outer membrane thiols may serve as a barrier restricting Hg(II) uptake (Dunham-Cheatham et al., 2014; Graham et al., 2012; Hu et al., 2013; Liu et al., 2016). An alternative view considers Hg(II)-RSH surface complexation as the initial step in the uptake process (An et al., 2019; Lin et al., 2014; Zhao et al., 2017). Adediran et al. (2019) proposed that Hg(II) may form ternary complexes involving membrane thiols and extracellular ligands. The letter suggestion find support in EXAFS measurements showing that only 5% of membrane thiols (inner + outer membranes) participate in bis-thiolate Hg(LMM-RS)_2_ coordination, whereas 95% form ternary species (Song et al., 2020). Similarly, Gutensohn (2023) found that 42% of outer membrane thiols take part in ternary rather than bis-thiolate complexation. We propose that the contrasting effects of outer membrane thiols, as reported in the literature, may be explained by a parallel formation of bis-thiolate and ternary Hg(II) coordinations at the cell surface. Bis-thiolate complexes may impede uptake, whereas ternary complexes may promote transfer, offering a mechanistic basis for the observed variability in Hg(II) uptake behaviors.

### 4.3 Periplasmic thiols

The periplasm contains 3.6 × 10^−11^ μmol cell^-1^ of thiols, representing about 20% of total thiols in the full culture (or ~30% of whole-cell thiols when the extracellular fraction is excluded). Its thiol density (140 μmol g^-1^ C) is lower than that of either inner or outer membrane, between which the periplasm is situated. During Hg(II) uptake, Hg(II) passes from the outer membrane into the periplasm before reaching the inner membrane, making the periplasm the aqueous intermediary between two solid-phase membranes. Its comparatively lower thiol density likely slows the transfer of Hg(II) to the inner membrane. This aligns with findings by Schaefer et al. (2014) and Wang et al. (2020) who showed that transport into the periplasm was the rate-limiting step, and higher Hg(II) uptake rates were observed in *G. sulfurreducens* spheroplasts (cells lacking outer membranes and retaining only the inner membrane) compared to intact whole-cells.

Further, the EXAFS indication on the formation of Hg-RSSR and β-HgS makes the periplasm particularly noteworthy. The presence of disulfides (RSSR) in the periplasm of *G. sulfurreducens* is expected, as the periplasm (in prokaryotes) is the primary site of disulfide-bond formation, a process essential for proper protein folding and stability, since proteins with reduced (free) cysteines are rapidly degraded (Bardwell et al., 1991; Sevier and Kaiser, 2002). In Gram-negative bacteria such as *E. coli*, the Dsb (disulfide-bond formation) proteins, including DsbA and DsbB, together with related periplasmic enzymes, catalyze thiol oxidation and generate RSSR species (Nakamoto and Bardwell, 2004; Ruiz et al., 2006). The small sulfide fraction detected by EXAFS (~13%, near the fitting uncertainty) may arise as a byproduct of disulfide-bond formation/degradation, and prior studies report sulfide production in *G. sulfurreducens* under specific metabolic conditions (e.g., when S^0^ serves as an electron acceptor) (Caccavo et al., 1994). Regardless of its origin, the periplasm provides a redox-active environment for Hg-S reactions that likely have an impact on Hg(II) internalization and transformation.

### 4.4 Inner membrane thiols

The inner membrane poses the highest thiol concentration, approximately 7 × 10^−11^ μmol cell^-1^, representing about 37% of total thiols in the full culture (or ~50% of whole-cell thiols when the extracellular fraction is excluded). The relatively high thiol density of the inner membrane (450 μmol g^-1^ C) further supports its role in cellular Hg(II) internalization. This interpretation is consistent with observations that spheroplasts of *G. sulfurreducens* accumulate more Hg and exhibit higher uptake rates than intact cells (Schaefer et al., 2014; Wang et al., 2020). These studies suggest that Hg(II) complexes such as Hg(Cys)_2_ or HgCl_2_ passively diffuse across the outer membrane and periplasm, followed by an active transport across the inner membrane into the cytoplasm.

A plausible explanation may relate to differences in thiol-binding behavior. In the outer membrane, where ~58% of thiols are capable of forming bis-thiolate complexes Hg(RS)_2_, Hg could become sequestered in this coordination state on outer membrane, thereby reducing opportunity for ligand exchange. In contrast, the inner membrane is predominantly (~95%) associated with ternary complex formation, Hg(RS)L, which might provide greater flexibility for ligand substitution and potentially support active transport into the cytoplasm. Nonetheless, this remains a hypothesis that requires experimental validation.

### 4.5 Cytoplasmic thiols

The cytoplasm contains 1.5 × 10^−11^ μmol cell^-1^ of thiols, accounting for about 8% of total thiols in the cell culture (or roughly 10% of whole-cell thiols when the extracellular fraction is excluded). The gradual increase in thiol abundance from the cytoplasm to the periplasm and then to the extracellular space is consistent with previous studies showing that LMM-RSH species are synthesized in the cytoplasm and subsequently secreted to the extracellular environment (Adediran et al., 2019). As cells progress through mid–exponential growth, cytoplasmic thiols decrease while extracellular thiols increase, as reported in Table 5. The cytoplasm exhibits a relatively low thiol density (40 μmol g^-1^ C), similar to that of the extracellular fraction.

Within the cytoplasm, thiols may function as competitive ligands that acquire Hg(II) from inner membrane complexes via ligand-exchange, thereby enabling Hg(II) entry into the cytoplasm. The comparatively low cytoplasmic thiol abundance and density may modulate the rate of Hg(II) transfer into the cytosol, influencing its intracellular availability for subsequent processes such as Hg(II) methylation.

### 4.6 Framework for modeling Hg(II) speciation and bioavailability

Previous studies have characterized the thermodynamic stability of Hg(II) complexes formed with LMM-RSH (Liem-Nguyen et al., 2017), natural organic matter associated thiols (Song et al., 2018), and membranes-associated thiols (Song et al., 2020), revealing comparable binding affinities (log *K* values). Building on this foundation, the detailed quantification of subcellular thiol distributions in this study provides a valuable framework for modeling Hg(II) speciation and bioavailability within one of model methylating bacteria, *G. sulfurreducens*. This information enables thermodynamic predictions of Hg(II) speciation and concentration across subcellular compartments, although the extent to which thermodynamic equilibrium, rather than kinetically constrained conditions, applies in living cells remains uncertain. Incorporation of subcellular thiol concentrations and their binding characteristics into biogeochemical models would enable model development to calculate Hg(II) uptake, intracellular partitioning, and methylation potential under varying environmental conditions.

Because many soft metals (e.g., Cd, Pb, Zn, Ag, and Cu) exhibit similar thiol-binding behavior, these reported results also provide a basis for understanding how other thiol-reactive metals may be partitioned and transformed within cells.

## 5 Conclusion

In conclusion, this study provides the first comprehensive characterization of subcellular thiol distribution in *G. sulfurreducens*, offering new information enabling insights on Hg(II) uptake and transformation mechanisms. Our findings highlight the potential role of thiol-mediated processes in regulating mercury speciation, bacterial uptake and internalization, and methylation at the subcellular level.

Based on the sub-cellular distribution of thiol groups and their densities, further *in situ* studies are required to advance the understanding of mechanisms behind Hg uptake, internalization and transformation under dynamic *in vivo* conditions. Specifically, experiments tracking Hg(II) transport and chemical speciation within subcellular compartments in living bacteria throughout the uptake and methylation process would advance our molecular level understanding. Additional investigation of membranes-associated mechanisms, particularly the role of inner membrane metal transporters and channels, is necessary to improve our understanding of Hg(II) internalization pathways.

## Data Availability

The original contributions presented in the study are publicly available. This data can be found here: https://doi.org/10.5878/qpef-2205.
