# Supplementary material for: Subcellular thiol functional group distribution in Geobacter sulfurreducens determined by Hg LIII-edge EXAFS
Source: Front Microbiol. 2026 Feb 4;16:1728775. doi: 10.3389/fmicb.2025.1728775 (PMC12915048; doi:10.3389/fmicb.2025.1728775)
Supplement: Supplementary file 1 [file Data_Sheet_1.pdf]

frontiers

Supplementary Material

This Supplementary Material contains 2 figures and 5 tables.

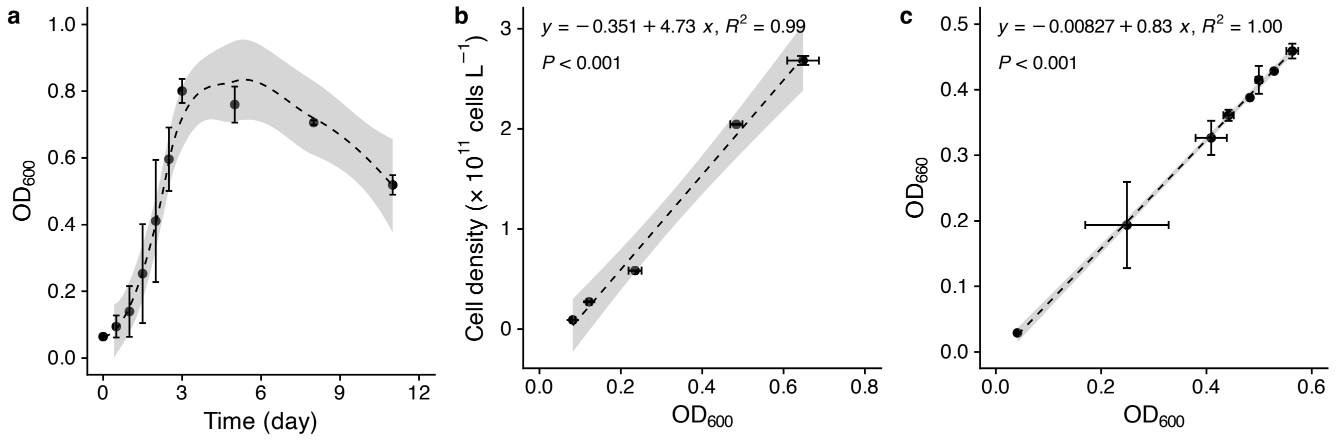

Figure S1: Growth of *G. sulfurreducens* and correlation between optical density and cell density. (a) Growth curve in standard medium, measured as OD_{600} over time. (b) Linear correlation between OD_{600} and cell density. (c) Linear correlation between OD_{600} and OD_{660}. Error bars indicate standard deviations from triplicate experiments.

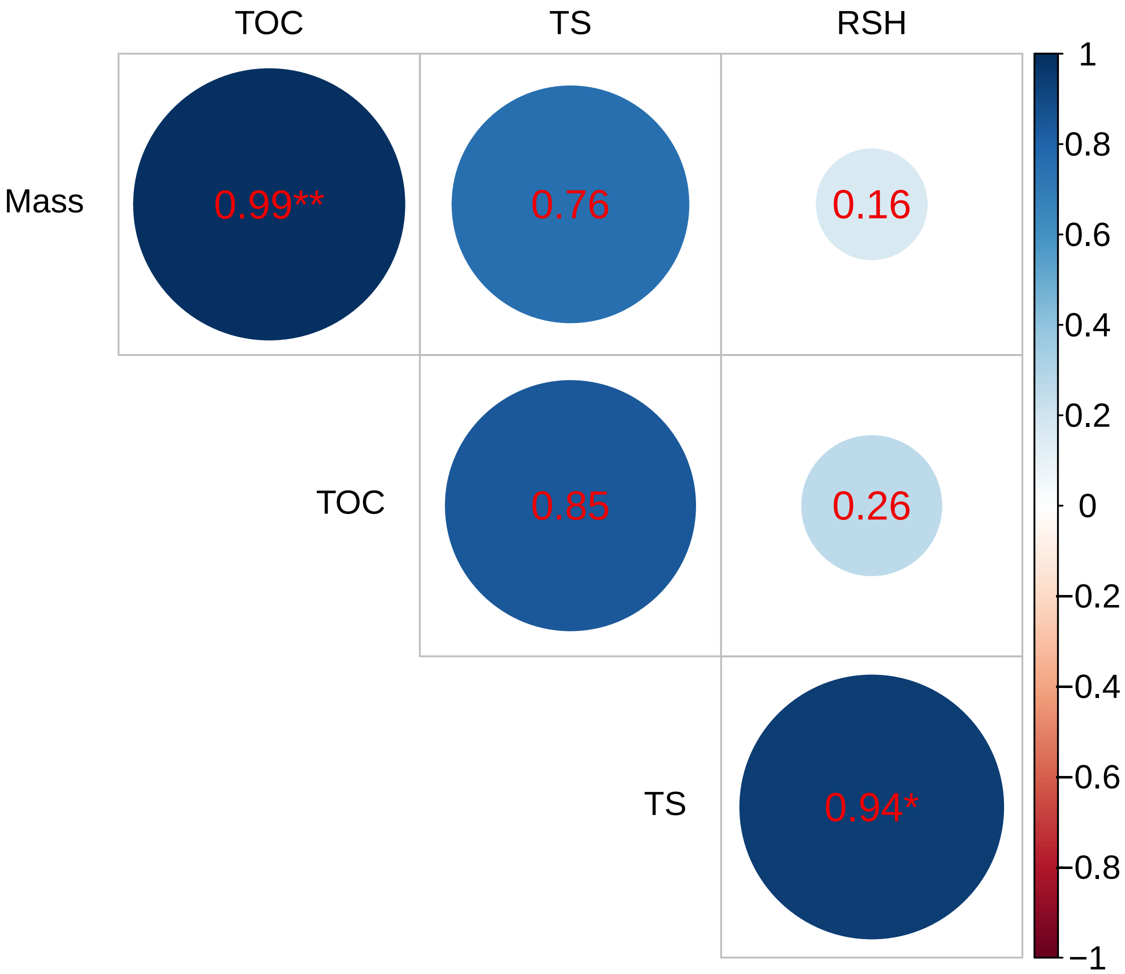

Figure S2: Pearson correlation matrix showing the relationships among subcellular dry mass (Mass, g $cell^{-1}$), total organic carbon (TOC, g $cell^{-1}$), total sulfur (TS, µmol $cell^{-1}$), and thiol concentration (RSH, µmol $cell^{-1}$). The color and size of the circles, along with the red numerical labels, indicate the strength and direction of the correlations. Asterisks denote significance levels: * $p < 0.05$ and ** $p < 0.01$.

Table S1: Initial Added and Final Hg(II) Concentrations and Corresponding Loss Percentages in Extracellular and Subcellular Fractions of *G. sulfurreducens* During Titration Experiments (mean ± SD; n = 3–10)

Sample	OD_{600}	Hg_{dded} (µmol g^{-1})	Hg_{final} (µmol g^{-1})	Hg Losses (%)
Ex-Mid-Std	0.51	0.62	0.23 ± 0.03	64 ± 5
Ex-Mid-Std	0.51	6.2	1.3 ± 0.4	79 ± 6
Ex-Mid-Std	0.51	61	49 ± 5	20 ± 8
Ex-Mid-Std	0.51	548	433 ± 11	21 ± 2
Ex-Mid-Std	0.60	0.07	0.06 ± 0.002	9 ± 3
Ex-Mid-Std	0.60	62	36 ± 0.3	43 ± 1
Ex-Late-Std	0.75	0.06	0.04 ± 0.01	33 ± 8
Ex-Late-Std	0.75	61	22 ± 2.1	64 ± 3
Ex-Late-Std	0.83	0.08	0.07 ± 0.01	14 ± 7
Ex-Late-Std	0.83	0.62	0.3 ± 0.04	51 ± 7
Ex-Late-Std	0.83	62	1.5 ± 0.3	75 ± 5
Ex-Late-Std	0.83	62	56 ± 12	9 ± 19
Ex-Late-Std	0.83	616	502 ± 25	18 ± 4
Ex-Mid-HS	0.58	0.06	0.04 ± 0.001	38 ± 2
Ex-Mid-HS	0.58	60	25 ± 5	59 ± 8
Cy-Mid-Std	0.51	0.37	0.31 ± 0.05	17 ± 14
Cy-Mid-Std	0.51	3.7	2.3 ± 0.2	39 ± 4
Cy-Mid-Std	0.51	37	36 ± 2.3	3 ± 6
Cy-Mid-Std	0.51	467	450 ± 22	4 ± 5
Cy-Mid-Std	0.60	0.1	0.06 ± 0.01	50 ± 5
Cy-Mid-Std	0.60	1.2	0.6 ± 0.1	55 ± 9
Cy-Mid-Std	0.60	3.6	2.4 ± 0.3	34 ± 7
Cy-Mid-Std	0.60	36	34 ± 8	4 ± 21
Cy-Late-Std	0.83	0.06	0.07 ± 0.01	0 ± 7.9
Cy-Late-Std	0.83	0.6	0.4 ± 0.02	34 ± 3
Cy-Late-Std	0.83	3.7	2.2 ± 0.1	40 ± 3
Cy-Late-Std	0.83	37	32 ± 5	14 ± 14
Cy-Late-Di	0.73	0.36	0.34 ± 0.03	7 ± 7
Cy-Late-Di	0.73	3.6	3.5 ± 0.6	4 ± 17
Cy-Mid-HS	0.58	0.37	0.35 ± 0.11	6 ± 29
Cy-Mid-HS	0.58	3.7	2.8 ± 0.2	24 ± 5

(continued on next page)

Sample	OD_{600}	Hg_{dded} ($\mu mol\,g^{-1}$)	Hg_{final} ($\mu mol\,g^{-1}$)	Hg Losses (%)
Cy-Mid-HS	0.58	63	46 ± 2	26 ± 3
Cy-Mid-HC	0.66	0.07	0.06 ± 0.01	10 ± 8
Cy-Mid-HC	0.66	0.67	0.31 ± 0.03	55 ± 4
Cy-Mid-HC	0.66	6.7	3.7 ± 0.6	45 ± 9
Cy-Mid-HC	0.66	61	44 ± 4	28 ± 6
Pe-Mid-Std	0.51	0.11	0.11 ± 0.02	0 ± 16
Pe-Mid-Std	0.51	1.1	0.8 ± 0.2	23 ± 22
Pe-Late-Std	0.83	0.11	0.12 ± 0.01	0 ± 6
Pe-Late-Std	0.83	1.1	0.9 ± 0.2	21 ± 16
Pe-Late-Std	0.83	11	8 ± 3	28 ± 29
WC-Late-Di	0.73	0.99	0.65 ± 0.02	34 ± 2
WC-Late-Di	0.73	9.9	8.5 ± 1.6	14 ± 16
WC-Late-Di	0.73	99	84 ± 4.4	15 ± 4
WC-Late-Di	0.73	986	673 ± 131	32 ± 13

Sample names follow the format "Subcellular Component-Growth Phase-Treatment", where:
Cy = Cytoplasm, Pe = Periplasm, Ex = Extracellular, Pe = Periplasm, WC = Whole-cell, IM = Inner membrane, OM = Outer membrane.
"Mid" refers to the middle-exponential phase (2 days), and "Late" refers to the late-exponential phase (3 days).
"Std" = Standard growth medium, "HC" = High-carbon medium (3× fumarate), "HS" = High-sulfur medium (10× sulfate), "Di" = Cell disruptor treatment.
OD_{600} represents optical density at 600 nm.
Hg_{add} denotes the initial Hg(II) concentration added; Hg_{final} refers to the final total Hg concentration measured after EXAFS experiments.
Gray-shaded rows indicate Hg(II) titration samples analyzed by EXAFS, while non-shaded rows correspond to Hg(II) titration samples without EXAFS analysis.

Table S2. Distribution of Dry Mass Across Extracellular and Subcellular Compartments of *G. sulfurreducens* (mean ± SD; n = 3)

**Subcellular Compartments**	**Protocol 1**[a]	**Protocol 2**[b]	**Average**[c]	**Distribution in whole-cell**[d]	**Distribution in cell culture**[e]
	($\times 10^{-12}$ g cell^{-1})			(%)	(%)
Extracellular	5.9 ± 0.5	6.2 ± 2.4	6.0 ± 1.7	n.a.	75 ± 27
Cytoplasm	1.4 ± 0.2	1.0 ± 0.2	1.2 ± 0.2	59 ± 13	15 ± 4
Periplasm	0.5 ± 0.05	n.d.	0.5 ± 0.1	25 ± 4	6 ± 1
Membranes	n.d.	0.3 ± 0.04	0.3 ± 0.04	15 ± 3	4 ± 1
Inner membrane	n.d.	0.24 ± 0.04	0.24 ± 0.04	12 ± 2	3 ± 1
Outer membrane	n.d.	0.06 ± 0.02	0.06 ± 0.02	3 ± 1	0.8 ± 0.3
Debris	n.d.	0.03 ± 0.02	0.03 ± 0.02	1 ± 0.9	0.4 ± 0.2
Whole-cell	1.5 ± 0.1	1.6 ± 0.4	1.56 ± 0.2	100 ± 14	25 ± 6
Recovery (%)*	n.a.	148 ± 40	130 ± 23		100 ± 27

[a] Values obtained using Protocol 1.

[b] Values obtained using Protocol 2.

[c] Averaged results from Protocols 1 and 2.

[d] Percentage of each subcellular fraction relative to the total whole-cell mass (excluding extracellular fractions).

[e] Percentage of each fraction relative to total biomass in the entire culture (including both whole-cell and extracellular fractions).

* Recovery (%) calculated as: (cytoplasm + periplasm + inner membrane + outer membrane + debris) / whole-cell × 100%.

n.a. = not available; n.d. = not determined. "Membranes" indicates pooled values when inner and outer membranes are not separated.

Table S3. Distribution of Total Organic Carbon (TOC) Across Extracellular and Subcellular Compartments of *G. sulfurreducens* (mean ± SD; n = 3)

Subcellular Compartment	Protocol 1[a] (× 10^{-13} g $cell^{-1}$)	Protocol 2[b] (× 10^{-13} g $cell^{-1}$)	Average[c] (× 10^{-13} g $cell^{-1}$)	Distribution in whole-cell[d] (%)	Distribution in cell culture[e] (%)	TOC per subcellular dry mass[f] (%)
Extracellular	19 ± 9	n.d.	19 ± 9	n.a.	69 ± 28	32 ± 18
Cytoplasm	4.1 ± 0.4	4.4 ± 0.8	4.3 ± 0.7	48 ± 11	15 ± 5	36 ± 0.9
Periplasm	0.9 ± 2	4.4	2.6 ± 1.4	30 ± 17	9 ± 6	53 ± 29
Membranes	5.7 ± 2.4	1.8 ± 0.2	1.8 ± 0.2	20 ± 4	6 ± 2	59 ± 11
Inner membrane	n.d.	1.6 ± 0.3	1.6 ± 0.2	18 ± 4	6 ± 1	67 ± 14
Outer membrane	n.d.	0.2 ± 0.06	0.2 ± 0.03	2 ± 0.5	0.7 ± 0.3	31 ± 10
Debris	n.d.	0.1 ± 0.08	0.1 ± 0.04	2 ± 0.5	0.5 ± 0.2	45 ± 3
Whole-cell	11 ± 1.2	8.3 ± 1.2	7.2 ± 1.0	100 ± 21	31 ± 8	46 ± 2
Recovery (%)*	n.a.	129 ± 16	123 ± 23		100 ± 29	

[a] TOC values obtained using Protocol 1.

[b] TOC values obtained using Protocol 2.

[c] Averaged TOC values from Protocols 1 and 2.

[d] Percentage of TOC relative to the whole-cell (excluding extracellular content).

[e] Percentage of TOC in each fraction relative to cell culture (including both whole-cell and extracellular TOC).

[f] Percentage of TOC relative to the dry mass of each individual subcellular compartment.

* Recovery (%) was calculated as: (cytoplasm + periplasm + inner membrane + outer membrane + debris) / whole-cell × 100%.

n.a. = not available; n.d. = not determined. "Membranes" indicates pooled values when inner and outer membranes are not separated.

Table S4. Distribution of Total Sulfur (TS) Across Extracellular and Subcellular Compartments of *G. sulfurreducens* (mean ± SD; n = 3)

**Subcellular Compartment**	**Protocol 1[a]**	**Protocol 2[b]**	**Average[c]**	**Distribution in whole-cell[d]**	**TS per subcellular dry mass[f]**
	(× 10^{-10} µmol cell^{-1})			(%)	(%)
Cytoplasm	1.1 ± 0.2	1.0 ± 0.2	1.0 ± 0.2	32 ± 7	0.28 ± 0.7
Periplasm	0.26 ± 0.04	0.21 ± 0.03	0.24 ± 0.04	7 ± 1.4	0.14 ± 0.01
Membrane	6.7 ± 1.7	1.6 ± 0.2	1.6 ± 0.2	51 ± 8	1.7 ± 0.3
Inner membrane	5.8 ± 1.7	1.6 ± 0.2	1.6 ± 0.2	49 ± 8	2.1 ± 0.4
Outer membrane	0.9 ± 0.3	0.07 ± 0.01	0.07 ± 0.01	2 ± 1	0.3 ± 0.1
Debris	n.d.	0.3 ± 0.2	0.3 ± 0.2	9 ± 7	3.2 ± 2.0
Whole-cell	7.9 ± 1.2	3.3 ± 0.7	4.2 ± 0.7	100 ± 13	0.85 ± 0.09
Recovery (%)[*]	n.a.	95 ± 18	77 ± 15		

[a] TS values obtained using Protocol 1.

[b] TS values obtained using Protocol 2.

[c] Average TS values from Protocols 1 and 2.

[d] Percentage of TS in the whole-cell (excluding the extracellular component).

[f] Percentage of TS relative to the dry mass of each individual subcellular compartment.

[*] Recovery (%) was calculated as: (cytoplasm + periplasm + inner membrane + outer membrane + debris) / whole-cell × 100%.

n.a. = not available; n.d. = not determined. "Membranes" indicates pooled values when inner and outer membranes are not separated.

Table S5: EXAFS Result Including 1st and 2nd Shell Fitting.

**Sample**	**OD_{600}**	**Hg_{final}**	**R_p**	**ΔE_0**	**Hg−Hg at $Hg(RS)_2$** 1st S shell, 2nd C(−RS) shell, MS (S−Hg−S), 2nd RSSR			**Hg−O/N at $Hg(RO/N)_2$** 1st O shell			**Hg−Hg at Hg^0** $Hg-Hg_1$, $Hg-Hg_2$, $Hg-Hg_3$, $Hg-Hg_4$			**Hg−Hg at HgS** 1st S shell, 2nd Hg shell, 2nd S shell		
					**CN**	**R**	**σ^2**	**CN**	**R**	**σ^2**	**CN**	**R**	**σ^2**	**CN**	**R**	**σ^2**
Cy-Mid-Std	0.51	0.3	4.5	5.6	1.62[c]	2.35	0.006									
					1.62[c]	3.48	0.015									
					1.62[c]	4.62	0.015									
					0.35	2.93[f]	0.003									
Cy-Mid-Std	0.51	2.2	8.4	4.3	0.65[c]	2.35	0.003	0.12	2.05	0.003						
					0.65[c]	3.3	0.011									
					0.65[c]	4.64	0.015									
Cy-Mid-Std	0.6	2.4	6.9	5.6	0.76[c]	2.35	0.003				1.02[c]	2.99[f]	0.003			
					0.76[c]	3.29	0.015				1.02[c]	3.46[f]	0.015			
					0.76[c]	4.63	0.015				1.02[c]	4.57[f]	0.015			
											1.02[c]	4.88[f]	0.015			
Cy-Late-Std	0.83	0.4	9.7	8.7	2.10[c]	2.35	0.004									
					2.10[c]	3.32	0.009									
					2.10[c]	4.67	0.015									
Cy-Late-Std	0.83	2.2	18	4.9	0.71[c]	2.37	0.003	0.31	2.05	0.003						
					0.71[c]	3.3	0.015									
					0.71[c]	4.59	0.015									
Cy-Mid-HC	0.66	0.3	5	7.7	1.97[c]	2.34	0.004									

(continued on next page)

Sample	OD_{600}	Hg_{final}	R_p	ΔE_0	Hg−Hg at $Hg(RS)_2$<br>1st S shell<br>2nd C(−RS) shell<br>MS (S−Hg−S)<br>2nd RSSR			Hg−O/N at $Hg(RO/N)_2$<br>1st O shell			Hg−Hg at Hg^0<br>$Hg-Hg_1$<br>$Hg-Hg_2$<br>$Hg-Hg_3$<br>$Hg-Hg_4$			Hg−Hg at HgS<br>1st S shell<br>2nd Hg shell<br>2nd S shell		
					**CN**	**R**	σ^2	**CN**	**R**	σ^2	**CN**	**R**	σ^2	**CN**	**R**	σ^2
					1.97[c]	3.32	0.015									
					1.97[c]	4.65	0.011									
Cy-Mid-HC	0.66	3.7	19	4.4	0.59[c]	2.41	0.003	0.28	2.11	0.003				0.15[c]	2.53[f]	0.005
					0.59[c]	3.3	0.015							0.45[c]	4.15[f]	0.013
					0.59[c]	4.44	0.015							0.45[c]	4.77[f]	0.015
Cy-Mid-HS	0.58	0.3	9.6	3.8	1.50[c]	2.36	0.004									
					1.50[c]	3.48	0.003									
					1.50[c]	4.62	0.009									
					0.47	2.98	0.003									
Cy-Mid-HS	0.58	2.8	8.6	7.7	0.57[c]	2.38	0.003	0.36	2.11	0.004	0.27[c]	2.99[f]	0.003			
					0.57[c]	3.3	0.007				0.27[c]	3.46[f]	0.015			
					0.57[c]	4.55	0.015				0.27[c]	4.57[f]	0.015			
											0.27[c]	4.88[f]	0.015			
Pe-Mid-Std	0.51	0.1	20	9.9	1.77[c]	2.35	0.003									
					1.77[c]	3.36	0.003									
					1.77[c]	4.7	0.015									
					0.4	2.85	0.015									
Pe-Mid-Std	0.51	0.8	35	11	0.53[c]	2.37	0.003							0.16[c]	2.53[f]	0.015
					0.53[c]	3.29	0.003							0.47[c]	4.15[f]	0.003
					0.53[c]	4.48	0.007							0.47[c]	4.77[f]	0.015
					1.99	2.86	0.015									

(continued on next page)

Sample	OD_{600}	Hg_{final}	R_p	ΔE_0	Hg−Hg at $Hg(RS)_2$<br>1st S shell<br>2nd C(−RS) shell<br>MS (S−Hg−S)<br>2nd RSSR			Hg−O/N at $Hg(RO/N)_2$<br>1st O shell			Hg−Hg at Hg^0<br>$Hg-Hg_1$<br>$Hg-Hg_2$<br>$Hg-Hg_3$<br>$Hg-Hg_4$			Hg−Hg at HgS<br>1st S shell<br>2nd Hg shell<br>2nd S shell		
					**CN**	**R**	σ^2	**CN**	**R**	σ^2	**CN**	**R**	σ^2	**CN**	**R**	σ^2
Pe-Late-Std	0.83	0.9	11.4	25	0.91[c]	2.36	0.003									
					0.91[c]	3.28	0.004									
					0.91[c]	4.5	0.015									
					2.57	2.87	0.015									
Ex-Mid-Std	0.51	0.2	14	6.7	1.88[c]	2.34	0.008									
					1.88[c]	3.3	0.013									
					1.88[c]	4.6	0.015									
Ex-Mid-Std	0.51	1.3	8.5	7	0.65[c]	2.36	0.003	0.46	2.06	0.015	1.38[c]	2.99[f]	0.003			
					0.65[c]	3.48	0.015				1.38[c]	3.46[f]	0.015			
					0.65[c]	4.61	0.015				1.38[c]	4.57[f]	0.011			
											1.38[c]	4.88[f]	0.015			
Ex-Late-Std	0.83	0.3	8.6	9.1	1.85[c]	2.35	0.003									
					1.85[c]	3.32	0.006									
					1.85[c]	4.67	0.011									
Ex-Late-Std	0.83	1.5	15	6.8	0.89[c]	2.36	0.004				2.01[c]	2.99[f]	0.003			
					0.89[c]	3.48	0.015				2.01[c]	3.46[f]	0.015			
					0.89[c]	4.64	0.015				2.01[c]	4.57[f]	0.013)			
											2.01[c]	4.88[f]	0.015			
WC-Late-Di	0.73	0.7	3.5	5.9	2.08[c]	2.35	0.005									
					2.08[c]	3.31	0.015									

(continued on next page)

Sample	OD_{600}	Hg_{final}	R_p	ΔE_0	Hg−Hg at $Hg(RS)_2$			Hg−O/N at $Hg(RO/N)_2$			Hg−Hg at Hg^0			Hg−Hg at HgS		
					1st S shell			1st O shell			$Hg-Hg_1$			1st S shell		
					2nd C(−RS) shell						$Hg-Hg_2$			2nd Hg shell		
					MS (S−Hg−S)						$Hg-Hg_3$			2nd S shell		
					2nd RSSR						$Hg-Hg_4$					
					CN	R	σ^2	CN	R	σ^2	CN	R	σ^2	CN	R	σ^2
					2.08[c]	4.63	0.015									
WC-Late-Di	0.73	8.5	0.9	8.1	2.18[c]	2.34	0.004									
					2.18[c]	3.3	0.012									
					2.18[c]	4.65	0.011									
WC-Late-Di	0.73	84.4	19	6.6	0.67[c]	2.42	0.003	0.3	2.05	0.003	0.88[c]	2.99[f]	0.015			
					0.67[c]	3.49	0.004				0.88[c]	3.46[f]	0.015			
					0.67[c]	4.88	0.015				0.88[c]	4.57[f]	0.015			
											0.88[c]	4.88[f]	0.014			

Sample names follow the format “Subcellular Component-Growth Phase-Treatment”, where:
Cy = Cytoplasm, Pe = Periplasm, Ex = Extracellular, Pe = Periplasm, WC = Whole-cell, IM = Inner membrane, OM = Outer membrane.
“Mid” refers to the middle-exponential phase (2 days), and “Late” refers to the late-exponential phase (3 days).
“Std” = Standard growth medium, “HC” = High-carbon medium (3× fumarate), “HS” = High-sulfur medium (10× sulfate), “Di” = Cell disruptor treatment.
OD_{600} represents the optical density measured at 600 nm.
Hg_{final} represents the final total Hg concentration determined after EXAFS experiments (µmol g^{-1}).
[c] denotes a correlated coordination number (CN), and [f] indicates that the bond distance (R) was fixed in the refinement.

EXAFS Fitting Parameters and Uncertainties:
Coordination Number (CN): 10%;
Bond Distance (R): 0.01–0.02 Å;
Edge Energy Shift (ΔE_0): 1–3 eV;
Debye–Waller Factor (σ^2): constrained between 0.003–0.015 $Å^2$;
Amplitude Reduction Factor (S_0^2): fixed at 0.9 for all samples.
R_p (%): Merit-of-Fit, calculated as $\sum(\text{model} - \text{experiment})^2 / \sum \text{experiment}^2$.
